# From thiol-subtilisin to omniligase: Design and structure of a broadly applicable peptide ligase

**DOI:** 10.1016/j.csbj.2021.02.002

**Published:** 2021-02-09

**Authors:** Ana Toplak, Eduardo F. Teixeira de Oliveira, Marcel Schmidt, Henriëtte J. Rozeboom, Hein J. Wijma, Linda K.M. Meekels, Rowin de Visser, Dick B. Janssen, Timo Nuijens

**Affiliations:** a EnzyPep B.V., Brightlands Campus Urmonderbaan 22, 6167 RD Geleen, The Netherlands; b Biotransformation and Biocatalysis, Groningen Biomolecular Sciences and Biotechnology Institute, University of Groningen, Nijenborgh 4, 9747 AG Groningen, The Netherlands

**Keywords:** Enzyme catalysis, Chemo-Enzymatic Peptide Synthesis (CEPS), Peptiligases, Omniligase-1

## Abstract

Omniligase-1 is a broadly applicable enzyme for peptide bond formation between an activated acyl donor peptide and a non-protected acyl acceptor peptide. The enzyme is derived from an earlier subtilisin variant called peptiligase by several rounds of protein engineering aimed at increasing synthetic yields and substrate range. To examine the contribution of individual mutations on S/H ratio and substrate scope in peptide synthesis, we selected peptiligase variant M222P/L217H as a starting enzyme and introduced successive mutations. Mutation A225N in the S1′ pocket and F189W of the S2′ pocket increased the synthesis to hydrolysis (S/H) ratio and overall coupling efficiency, whereas the I107V mutation was added to S4 pocket to increase the reaction rate. The final omniligase variants appeared to have a very broad substrate range, coupling more than 250 peptides in a 400-member library of acyl acceptors, as indicated by a high-throughput FRET assay. Crystal structures and computational modelling could rationalize the exceptional properties of omniligase-1 in peptide synthesis

## Graphical abstract

**Figure:**
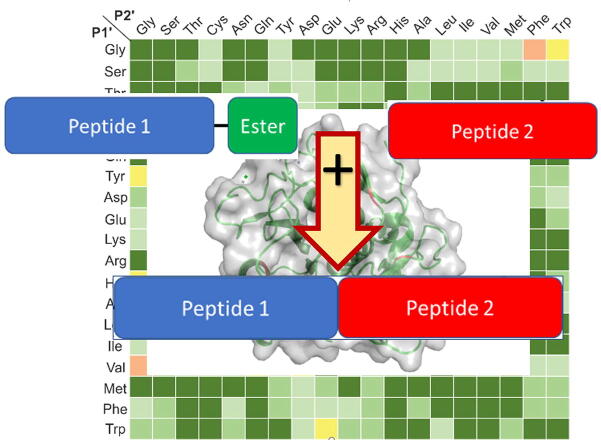

## 1 Introduction

The growing interest in large and complex peptides for pharmaceutical development has stimulated research on peptide synthesis and modification during the past decades [1]. Of particular interest are methods for the synthesis of larger peptides by formation of peptide bonds between chemically synthesized fragments [2]. Enzyme-mediated peptide coupling and modification methods can often offer great advantages over chemical procedures due to their excellent regio- and chemoselectivity and progress under mild reaction conditions in the absence of epimerization. However, only very few enzymes have been found in nature that catalyse ATP-independent peptide ligation, such as sortase [3], [4] and butelase [5], [6]. ATP-dependent reactions are also known, but only for the synthesis of small peptides [7], [8]. Yet the use of enzymes for peptide coupling and modification has been limited due to poor catalytic activity, narrow peptide selectivity or expression constraints [9]. Triggered by the need for practical coupling enzymes, it has been attempted to apply and engineer proteases, which naturally catalyse hydrolysis of peptide bonds. Protein engineering can alter the catalytic properties of serine hydrolases to make them suitable for the reverse (synthetic) reaction under kinetically controlled conditions[9], [10]. The coupling reactions can be carried out in aqueous medium, which is attractive in view of substrate solubility, but require enzymes that prefer a nucleophilic acyl acceptor over water for the cleavage of the covalent acyl-enzyme intermediate. An important goal is the engineering of enzymes with improved synthesis to hydrolysis ratios in peptide coupling reactions that employ C-terminally activated peptide fragments as acyl donor.

More than 50 years ago, the active site serine residue of the common serine protease subtilisin was chemically converted to a cysteine (S221C), resulting in an enzyme called thiol-subtilisin [11] which was later on applied in chemo-enzymatic peptide synthesis (CEPS) [12]. Although the change from –OH to –SH is relatively small, the effect on the catalytic properties is large since the covalent thioester acyl-enzyme intermediate is relatively more prone to cleavage by an amine nucleophile than the corresponding oxo ester, which is more sensitive to hydrolysis by water [13]. This principle was further exploited by the creation of seleno-subtilisin, an enzyme that is over 14,000 fold better in amide synthesis compared to the wild-type [14], [15]. However, the activity of both thiol- and seleno-subtilisin is drastically reduced as compared to the wild-type activity of subtilisin [14]. A few decades later, when protein engineering via mutagenesis became feasible, Wells and co-workers discovered that the activity of thiol-subtilisin could be restored by the introduction of a second mutation, i.e. P225A, that reduced the steric crowding in the active site created by the slightly more spacious thiol group compared to the wild-type hydroxy moiety. They created the S221C/P225A double mutant termed subtiligase, and showed it could couple a peptide *C*-terminal ester (acyl-donor) to a peptide bearing a free *N*-terminal amine (acyl-acceptor) in an aqueous environment. Although promising, average ligation yields with subtiligase were 60–70% and 10-fold excess of acyl-acceptor was used[13], [16], which is not good enough for industrial applicability. Besides that, subtiligase has a Ca^2+^ binding domain required for folding, which makes it a relatively unstable enzyme. Triggered by these stability issues, we incorporated the S221C and P225A mutations into a hyperstable (18 mutations and one disulfide bridge) calcium-independent (deletion of Ca^2+^-binding loop) subtilisin variant discovered by Bryan et. al (BS149) [17]. Besides the improved stability, the newly created enzyme, called peptiligase [18] (Ptl), proved to give a more than 2-fold higher synthesis over hydrolysis (S/H) ratio as compared to subtiligase.

Although peptiligase showed improved catalytic properties compared to the earlier subtiligase, a remaining limitation of Ptl is the narrow acyl acceptor substrate scope at the S1′ and S2′ pockets. In particular, only small amino acids such as Gly, Ala and Ser are well accepted in the S1′ pocket, thus limiting the ligation possibilities. Therefore, we set out to improve the substrate acceptance of Ptl by further protein engineering. By making structure-guided site-evaluation libraries at positions M222 and L217 of the S1′ pocket, we were able to create a toolbox of ligases with different substrate specificities [19]. Several of the Ptl-derived variants had a divergent preference for specific amino acids side-chain functionalities, such as positive vs. negative charge, large vs. small or polar vs. hydrophobic. Gratifyingly, some mutations also substantially increased the overall substrate scope, i.e. all 19 different P1′ amino acids (except Pro) were coupled with higher efficiency compared to the wild-type Ptl. Especially the Ptl variants with mutations M222G/L217F and M222P/L217H proved to have a much broader substrate scope and improved S/H ratio compared to the parent peptiligase. To make chemoenzymatic peptide synthesis (CEPS) economically attractive, equimolar nucleophile concentrations should preferably be used, and the unwanted loss of acyl-donor substrate due to chemical or enzymatic hydrolysis needs to be minimized. This requires enzymes that have high activity in the conversion of the somewhat labile acyl donor to acyl-enzyme intermediate and quickly react with the nucleophilic acyl acceptor, resulting in high coupling efficiencies.

Building on the positive results of the site-evaluation libraries of the S1′ pocket, we further examined the S1′ and S2′ pockets via rational design and site-directed mutagenesis, which led to the discovery of a broadly applicable ligase termed omniligase-1 (Oml-1).[21] To obtain this enzyme, we selected peptiligase variant M222P/L217H as a starting enzyme and examined the effect of mutations at position A225 of the S1′ pocket and of F189 of the S2′pocket on the coupling efficiency. To increase the reaction rate, mutation I107V was incorporated. Mutations having a positive effect were combined to give Oml-1. This enzyme was used for the synthesis of pharmaceutical peptides, conjugation of tags or polymers to proteins [20] and for the head-to-tail cyclisation of peptides[21], [22], [23], [24], [25], giving access to folded cyclotides. The ligation reactions were also combined with chemical ligation technologies, e.g. for the preparation of tetracyclic peptides [26], [27]. Most recently, it was shown that the Oml-1 coupling reactions could be applied in an industrial setting to access pharmaceutical peptides in a green manner [28].

The attractive features of Oml-1 raise the question which mutations are responsible for its exceptional synthetic performance and which structural features of the protein influence recognition of peptide substrates in the acyl-donor and acyl-acceptor binding sites. Also, the observation that the S/H ratio is improved due to unexpected mutations at position 225 asks for a structural explanation. To address these issues, we explored the catalytic profile and crystal structures of omniligase and related peptiligase variants. For substrate profiling we developed a high-throughput fluorescence-based screening assay and the substrate spectrum was evaluated computationally by Rosetta docking simulations. The results identify residues and interactions contributing to differential substrate acceptance and improved synthetic performance.

## 2 Materials and methods

### 2.1 Construction and expression of peptiligase variants

Peptiligase variants were prepared either by gene synthesis at GenScript or by QuikChange site-directed mutagenesis using an *E. coli*-*B. subtilis* shuttle vector. The mutations were confirmed by DNA sequencing by GATC (now Eurofins Genomics). Expression and enzyme purification were performed as described previously [19].

### 2.2 X-ray crystal structure determination of omniligase variants

All omniligase mutants were further purified after the His-tag purification step by gel filtration using a Superdex 75 HR10/30 column (Cytiva), equilibrated with 20 mM HEPES buffer, pH 7.3, containing 150 mM NaCl. Omniligase fractions were pooled and concentrated to 10 mg mL^−1^ using a Vivaspin-10 K filter unit (Sartorius). Dynamic light scattering experiments were performed using a DynaPro Nanostar instrument (Wyatt Technology Corporation, Santa Barbara, CA, USA) at 20 °C. Dynamic light scattering data were processed and analyzed using Dynamics software (Wyatt Technology Corporation, Santa Barbara, CA, USA) and apparent molecular masses of ca. 30 kDa were deduced with a polydispersity of <20%.

Crystallization trials were performed in 96-well MRC 2 well plates (Swissci AG, High Wycombe, UK), using a Mosquito crystallization robot (SPT Labtech Ltd. Melbourn, UK) with commercially available screening matrices. Droplets containing reservoir solution (75–125 nL) and protein solution (125–75 nL) were incubated against 50 µL of each reservoir solution at 21 °C.

Pre-1 crystals could be grown from ammonium sulfate conditions in several screens. A large Pre-1 crystal grew from an optimization screen with 1.4 M MgSO_4_ and 0.1 M MES pH 6.5. A Pre-2 crystal grew from JCSG^+^ condition E9, 1.6 M MgSO_4_, 0.1 M MES pH 6.5. A Pre-3 crystal grew form JCSG^+^ condition G2, 20% polyacrylic acid 5100, pH 7.5. Tiny crystals of Pre-4 supplemented with an eglin C fragment (Ac-LPEGSPVTLDLRY-NH_2_, UniProtKB P01051 residues 37–49) grew from Index condition C2 after 2 months of incubation. Optimization in hanging drops with 1.2–1.8 M ammonium tartrate, pH 7.0, yielded after 8 months small crystals large enough for X-ray diffraction. For Pre-5 many microcrystals grew in the optimization screen but only with the addition of the eglin C fragment a larger bipyramidal-like Pre-5 crystal was obtained after a few weeks of incubation from 1.2 M (NH_4_)_2_SO_4_, 0.1 M MES pH 6.0 and 0.25% PEG 3350. Initial Pre-6 crystals grew from Index screen condition E11. Optimization was performed with 20% polyacrylic acid 5100, 0.1 M HEPES, pH 7.5, in hanging drop.

Prior to data collection, single Pre-1, Pre-2, Pre-4 and Pre-5 crystals were briefly soaked in mother liquor with addition of 25% glycerol as cryoprotectant. The Pre3 and Pre6 crystals were transferred to 30% polyacrylic acid which serves as cryoprotectant. X-ray diffraction data were collected on an in-house MarDTB Goniostat System using Cu-Kα radiation from a Bruker MicrostarH rotating-anode generator equipped with HeliosMX mirrors. Intensity data were processed using XDS [29] and the CCP4 package [30]. A summary of data collection statistics is given in Table S3.

Molecular replacement for the first mutant (Pre-1) was performed using PHASER [31] with the thymoligase crystal structure (PDB code 5OX2) [32] and for later structures with the determined omniligase mutant. The models were refined using REFMAC5 [33] and COOT [34] was used for manual rebuilding and map inspection. All mutations designed in the constructs were confirmed by electron density.

The quality of the model was analyzed using MolProbity [35]. Figures were prepared using PyMOL (Schrödinger LLC) [36]. Atomic coordinates and experimental structure factor amplitudes for Pre-1, Pre-2, Pre-3, Pre-4, Pre-5 and Pre-6 have been deposited in the RCSB Protein Data Bank (Table S3) as PDB entries 7AM3, 7AM4, 7AM5, 7AM6, 7AM7 and 7AM8, respectively.

### 2.3 Peptide substrate synthesis and enzyme screening

Solid phase peptide synthesis (SPPS) protocols used to synthetize peptide substrates are described in the Supplementary Information.

The screening of S1′ and S2′ mutant enzyme libraries was performed using the following substrates: Ac-DFSKL-Cam-L-OH and H-ALR-NH_2_ for the S1′ enzyme library and Ac-DFSKL-Cam-L-OH and H-A-Xxx-LR-NH_2_ for screening 20 P2′ acyl acceptor fragments with 20 enzymes of the S2′ (F189X) library. To 20 µL of an aqueous solution containing both respective fragments (acyl donor: 10 mM, acyl acceptor: 15 mM) 20 µL of 1 M tricine buffer, pH 8.5, supplemented with TCEP (3.5 mM) were added. Next, 0.4 µg (15 pmol) of the respective enzyme variant was added to initiate the reaction, which was allowed to proceed at room temperature. After 30 min a 10 µL sample of the reaction mixture was quenched with 150 µL of a 2/98 (v/v) mixture of methylsulfonic acid/water, followed by analysis by HPLC-MS. For quantification the peak areas of starting material, ligation product and ester hydrolysis product were integrated. Percentage synthesis is defined as the peak area of synthetic product formed divided by the total sum of peak areas. S/H ratio is the ration between the peak area of synthetic product divided by the peak area of ester hydrolysis product.

The enzymes of the Pro225X library were tested in synthesis reactions using 3.3 mM nucleophile (H-SLR-NH_2_) and 8.3 mM acyl donor (Ac-DFSKL-OCam) in 0.08 M phosphate buffer, pH 8.0, with 1 mL total volume. To the reaction mixture 5.5 μg of enzyme was added followed by incubation for 30 min with shaking at room temperature. The reaction was stopped by quenching with 1% v/v methanesulfonic acid (MSA) in water to a final 1:3 vol ratio and the mixture was analyzed on LC-MS as described above.

### 2.4 FRET-based substrate screening

For testing the nucleophile (S1′ and S2′) acceptance scope of the respective peptiligase variants the reactivity of Abz-KFTKL-Cam-L-OH with the 400 peptide containing amine library (H-Xxx-Yyy-KK(Dnp)K-OH) was measured in a time-resolved fashion using a multi-well plate UV/VIS fluorescent reader. When ligation occurs the fluorescence of the Abz (aminobenzoic acid) group is quenched by Dnp (dinitrophenyl). Upon ligation a decrease of fluorescence signal due to FRET occurred, which offered a method for estimating the coupling yield. Acyl donor substrates were used at a concentration of 1.25 mM and acyl acceptor substrates at a concentration of 3.75 mM based on the molecular weight of the corresponding peptide trifluoroacetic acid (TFA) salts. Reactions were performed in 200 mM tricine buffer, pH 8.5, and followed by measuring the fluorescence at λ = 420 nm (excitation at λ = 320 nm) every minute for a total duration of 1 h. The amount of enzyme used varied between approx. 0.25 µM and 1.25 µM. In order to determine the optimal amount of enzyme used for full conversion of substrate H-ALKK(Dnp)-K-OH within 30 min for each variant a pre-screening test was performed with varying amounts of enzyme. Based on the results obtained with omniligase-1 two good (H-ALKK(Dnp)K-OH, H-DLKK(Dnp)K-OH), two average (H-RLKK(Dnp)K-OH, H-SSKK(Dnp)K-OH), and two bad (H-EIKK(Dnp)K-OH, H-QVKK(Dnp)K-OH) acyl acceptor substrates were chosen for the pre-screening.

### 2.5 Computational modelling

The changes in enzyme stability upon introduction of point mutations in peptiligase variants were predicted using FoldX and Rosetta_ddg [37], [38]. For FoldX, the standard protocol was used with 5 repeat calculations of which the results were averaged. For Rosetta, the standard protocols as described in row 3 and row 6 of Table 1 by Kellog *et al.* [38] were used. All three protocols were shown to give good prediction of stability changes without allowing the backbone atoms to shift position. Strain calculations were performed using Yasara [39] according to a published protocol [40]. For the strain calculations, an energy minimization was carried out, using the Amber ff14SB force field [41], while fixing the backbone atoms in place but allowing all other atoms to move. This allowed to relieve local clashes without dissipating local strain caused by unadapted backbone positions. The reported van der Waals energies were calculated with the same Amber ff14SB force field.

**Table 1:** Effect of 225X mutations on product yield and S/H ratio.

Ptl M222P/L217H/A225X variant	Synthesis (%)	S/H ratio
A225N	88	7.3
A225D	87	6.7
A225S	85	6.1
A225C	84	5.3
A225G	74	4.6
A225A	67	4.5
A225P	6	0.2

The Rosetta Molecular Modeling program (build 2019.35.60890) [42] was used to calculate binding modes and interaction energies for complexes of Oml-1 with peptide substrates. For this, we built an initial structure from enzyme variant Pre-6 with the conformation of the peptide backbone from P5 to P3′ (peptide DFSKL-P1′-P2′-K) adopted from the average position of the ligand backbones found in X-ray structures of subtilisin-peptide complexes (1CSE, 1LW6, 1OYV, 1R0R, 1SBN, 1SIB, 1SPB, 1TM1, 1TM3, 1TM4, 1TM5, 1TM7, 1TMG, 1TO1, 1TO2, 1V5I, 1Y1K, 1Y33, 1Y34, 1Y3B, 1Y3C, 1Y3D, 1Y3F, 1Y48, 1Y4A, 1Y4D, 1YU6, 2SEC, 2SIC, 2SNI, 3BGO, 3CNQ, 3CO0, 3SIC, 5OX2, 5SIC). In the resulting structure we designed the required mutations to generate Oml-1 in complex with the different peptide products by varying amino acids in the peptide. For that the Rosetta “backrub” protocol [43], [44] was used to generate approximately 150 models for each Oml-1-peptide complex. These protein-peptide conformations were refined using the Rosetta FlexPepDock high-resolution minimization protocol [45]. The structures were visually inspected to guarantee that the active site residues are not displaced and exposed to the solvent. To select only peptide binding modes that are close to reactive conformations, three geometric constrains involving Asp32, His64, Asn155, Cys221 and the P1 amino acid were implemented (see Supplementary Information). Finally, the structures that passed these geometric criteria were ranked according to FlexPepDock energy terms reweighted score “reweighted_sc”, interface score “I_sc” and peptide score “pep_sc”. “I_sc” corresponds to the sum of energies contributed by interactions at the interface between Oml-1 residues and peptide residues. We observed that the average of the lowest 5 conformations (whenever 5 conformations were available) according to “I_sc” could distinguish the best peptides from the worst peptides, thus this average was used to rank the different peptides.

## 3 Results and discussion

### 3.1 Engineering the S1′ pocket

**Fig. 1:**
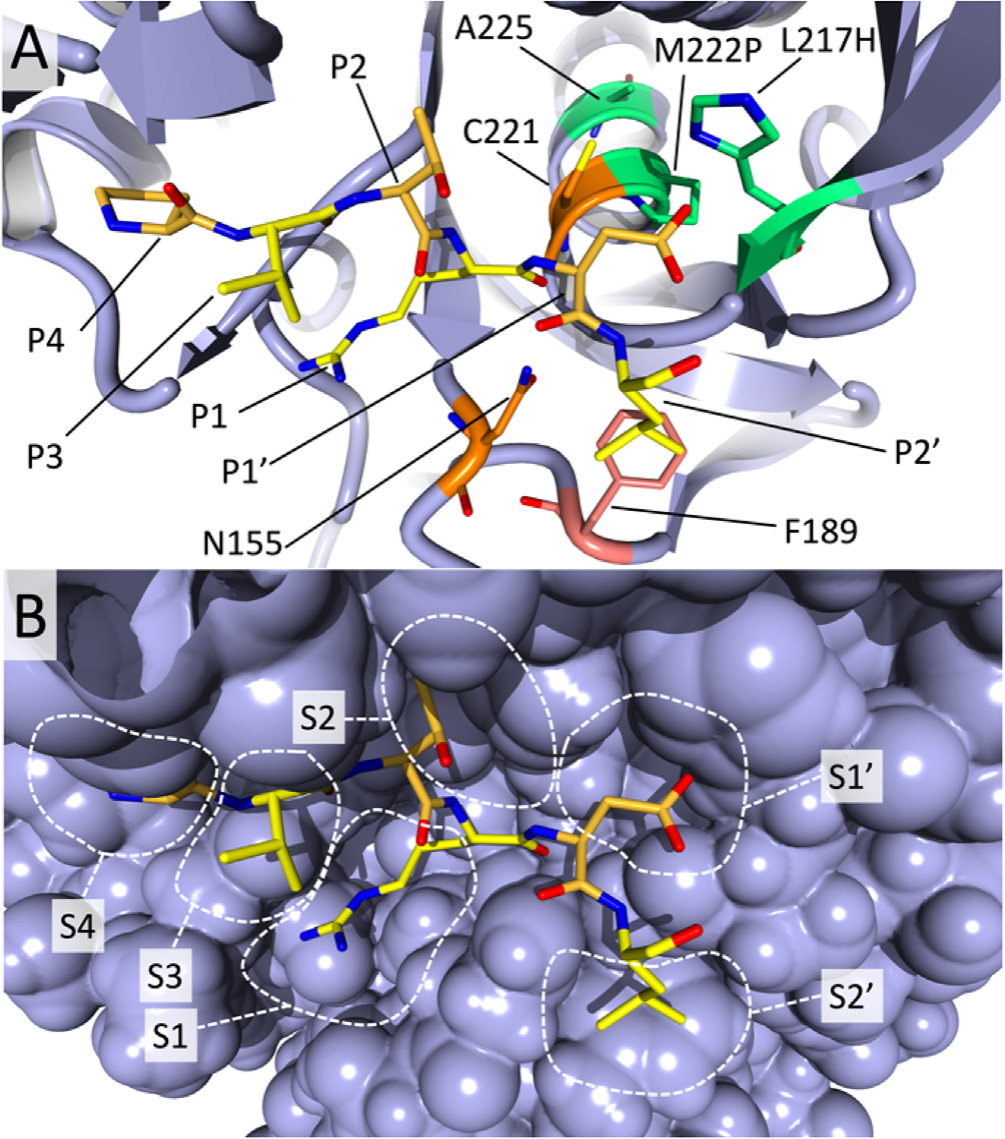
Hybrid model of peptiligase variant Ptl M222P/L217H with a fragment of eglin inhibitor variant (PVTRDL) bound into the S4-S2′ pockets. The interacting positions of the peptide (Pro-Val-Thr-Arg-Asp-Leu) are shown in different shades of yellow to facilitate distinction. A) The catalytically important residues Cys221 and Asn155 (the latter is part of the oxyanion hole) are shown in orange. The residues of the S1′ binding pocket involved in the contacts with the substrate P1′ residue are highlighted in light green (Pro222, Ala225 and His217). The S2′ pocket residue (Phe189) that was subjected to mutagenesis because it is in contact with the substrate P2′ (Leu) is shown in salmon. B) The position of the sub-pockets S4 to S2′ is illustrated by showing the protein in a van der Waals sphere surface. (For interpretation of the references to colour in this figure legend, the reader is referred to the web version of this article.)

To develop a broad-substrate spectrum peptiligase variant, we set out to further expand the acyl acceptor substrate scope of the earlier M222P/L217H variant of peptiligase [19]. First, to identify target positions, we constructed a model of this template enzyme using a calcium-independent subtilisin BPN' (pdb 1GNV [46]) and the subtilisin-eglin complex (pdb 1SBN) [47] as templates in which the mutations M222P and L217H were modeled [32]. Inspection of the model revealed several residues forming the S4-S2′ subsites that are in close contact with the side chains of the eglin inhibitor. Of the latter, the segment PVTRDL (residues 42–47) clearly interact with the protease P4-P2′ pockets (Fig. 1). Mutations at the positions shaping the S1′ and S2′ subsites were expected to influence the binding of the acyl acceptor fragments during peptide coupling and thus the range of amino acids accepted as a nucleophile in the cleavage of the covalent thioester intermediate and thereby lead to synthesis.

Based on inspection of the S1′ pocket in the model we targeted position Pro225. Wells and coworkers described mutation P225A and found this to be the sole substitution at position 225 that retains catalytic activity [13]. More recent subtiligase substrate engineering studies do not indicate otherwise [48]. Indeed, considering that amino acid 225 is located in an α-helix (amino acids 219–237) close to the active site and the possibility that the Ala225 amide proton forms a hydrogen bond with the oxygen of the nucleophilic residue Cys221, an effect of mutating position 225 on the catalytic performance of the enzyme is expected. However, the model of Ptl-M222P/L217H did not indicate why all substitutions should result in inactive enzyme and therefore we reexamined mutations at this position.

A complete site-directed 225X library (X = any proteinogenic amino acid) of Ptl-M222P/L217H was constructed. All enzyme variants could be produced in the usual *B. subtilis* expression system, although 225K, 225Y, 225M and 225W were obtained with low yields. The enzymes were tested in synthesis reactions with model peptides as described in Section 2.3. The results showed that not only P225A, but in total 14 out of 20 tested variants performed better compared to Ptl-M222P/L217H carrying the wild-type 225P. Surprisingly, this included five variants that outperformed the 225A variant in terms of S/H ratio. Clearly, small and polar residues that can form multiple hydrogen bonds, such as Asn, Asp, Ser, Cys, and also Gly gave better synthetic performance than Ala of the 225A parent (Table 1/S1).

We hypothesized that there is an optimum in the size and electronic properties for the amino acid at position 225. An explanation for the improved performance of mutants at this position is not apparent from the structural model. Using the Ptl-M222P/L217H/A225N with an almost doubled S/H ration as the new template, we next targeted the P2′ binding site.

### 3.2 Mutating Phe189 in the S2′ pocket

The S2′ subsite represents a hydrophobic surface flanked by Phe189, which according to the model has hydrophobic interactions with the P2′ Leu of the crystal bound eglin (Fig. 1). Accordingly, Phe189 was expected to influence the S2′ acyl acceptor substrate scope and was selected as a primary target for mutagenesis. After constructing a comprehensive site-directed library of Ptl M222P/L217H/F189X (X = all proteinogenic amino acids), the P2′ substrate scope of each of the 20 variants was mapped using a coupling assay with the model acyl donor Ac-DFSKL-OCam-Leu-OH to an acyl acceptor library of 20 variants of the tripeptide H-A-Xxx-R-NH_2_ (Xxx = all 20 proteinogenic amino acids).

The screening results showed that the hydrophobic residues Phe or Trp at position 189 give clearly enhanced synthetic yields (Fig. S1) and S/H ratios. Mutation F189W, in particular, appeared to have the best performance compared to the enzyme with F, Y or H at position 189. Examination of the F189W mutation in the structural model revealed increased hydrophobic interactions with the eglin P2′ Leu residue as compared to the wild-type Phe189. Surprisingly, after the introduction of F189W mutation even basic (R, K) as well as polar (Q, N) residues at position P2′ in H-A-Xxx-R-NH_2_ were increasingly well accepted as the amino acid to fit in the S2′ pocket. Also acceptance of Trp in position P2′ was significantly increased.

### 3.3 Screening a substrate library by FRET

Clearly, both mutations A225N and F189W appeared to be crucial to improve synthetic performance and broaden the substrate scope of Ptl-M222P/L217H (Fig. S2) [19]. Before exploring the substrate range of this combination mutant, we introduced an additional substitution. Literature data [49] suggest that the S4 pocket mutation I107V increases reaction rates, so it was also included, creating variant Ptl-I107V/F189W/L217H/M222P/A225N, and in view of its broad substrate scope (see below) we termed the enzyme omniligase-1, or Oml-1.

**Fig. 2:**
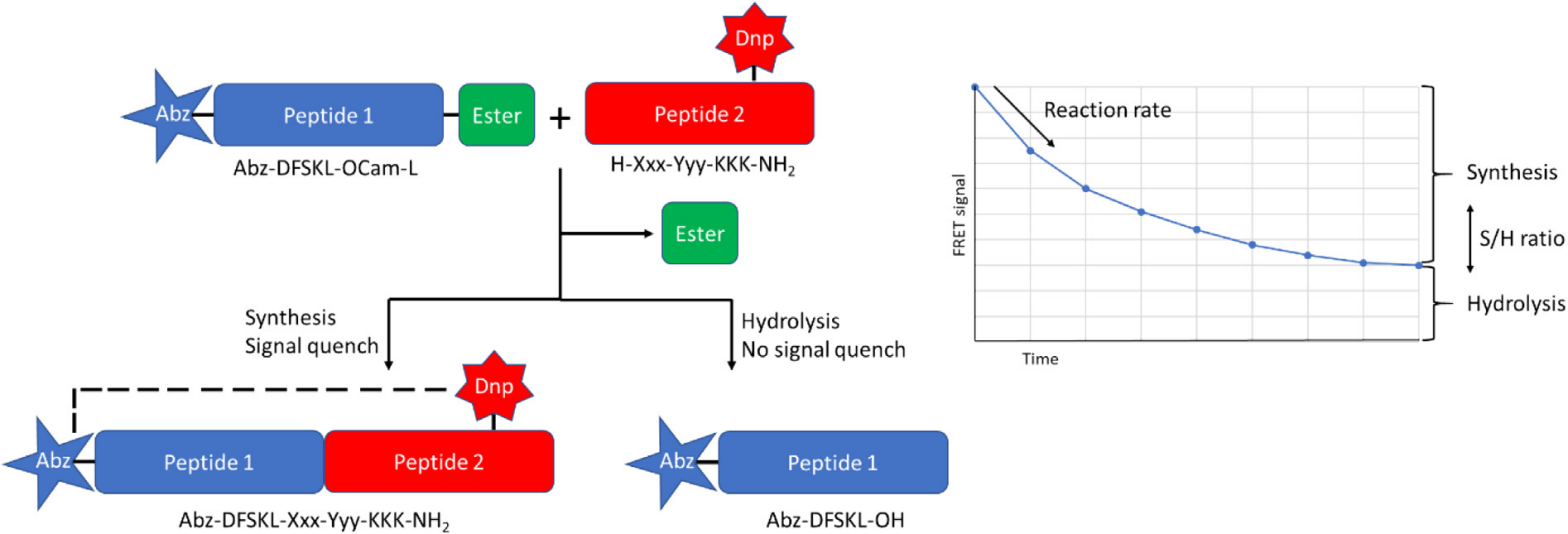
. FRET based screening of Ptl variants. Product formation leads to quenching of the fluorescence signal.

To rapidly screen the full P1′ + P2′ substrate scope of the Oml-1 derivative of peptiligase, a FRET (Förster resonance energy transfer) based screening assay was developed (Fig. 2). In these assays, a 2-aminobenzoyl (Abz) functionalized peptide ester (Abz-DFSKL-OCam-L) was coupled to a 2,4-dinitrophenyl (Dnp)-modified acyl acceptor library of 400 different fluorescently labeled peptides (H-Xxx-Yyy-Lys-Lys(Dnp)-Lys-NH_2_). Only product formation by peptide coupling leads to FRET quenching of the fluorescence signal, whereas a lack of activity and acyl donor hydrolysis both do not lead to signal decrease. By performing the reactions in multi-well plates, the reaction rates and S/H ratios could be determined for all substrates. Using a 96 well plate format, all 400 different coupling reactions could be performed and analyzed within a few hours.

**Fig. 3:**
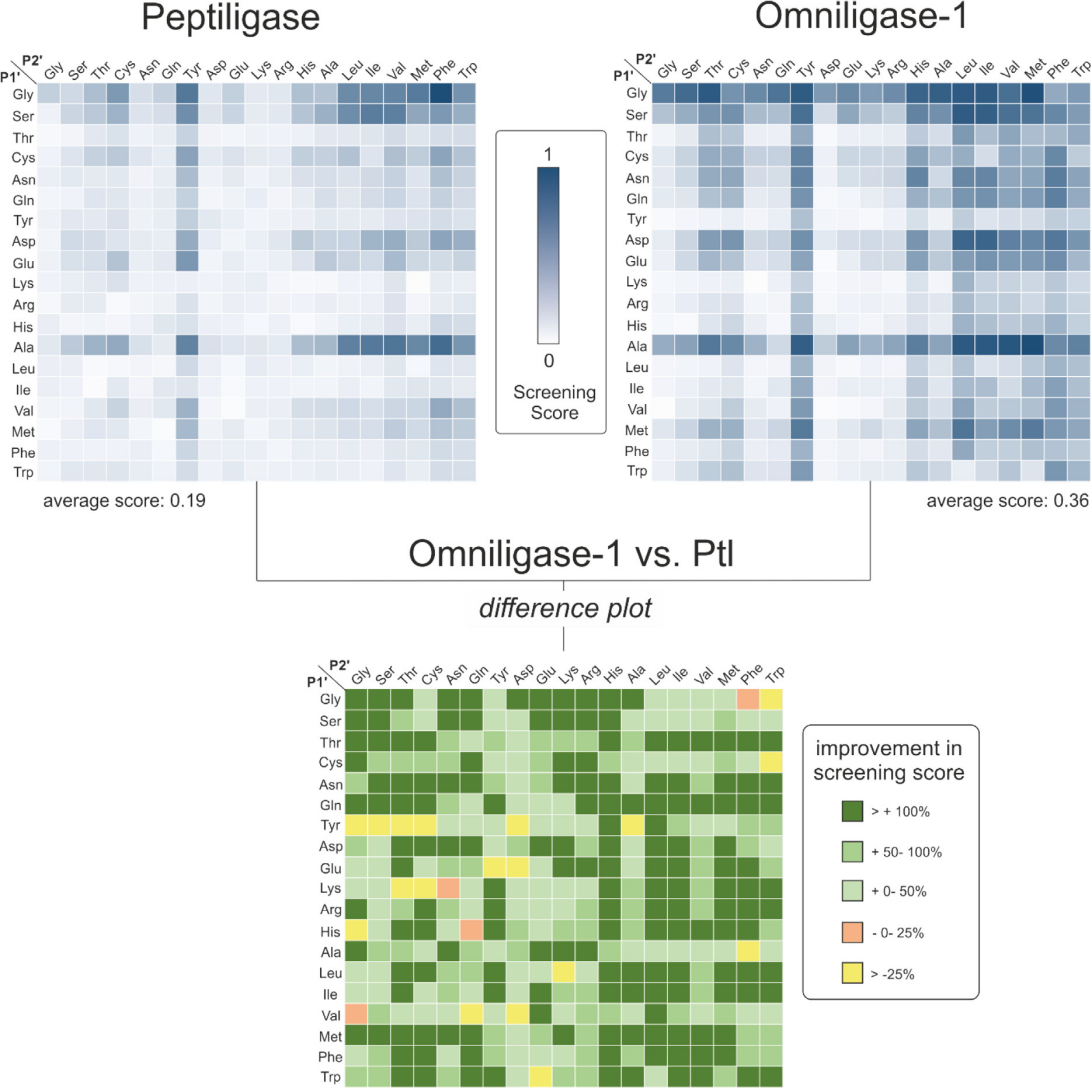
Screening peptide coupling activities of peptiligase and Oml-1 with a 400 acyl acceptor library using FRET assays. For testing the nucleophile acceptor scope (or substrate range at the S1′ and S2′ pockets) reactions were done in multi-well plates containing Abz-KFTKL-Cam-L-OH (fluorescent acyl donor) and a 400 peptide library of H-Xxx-Yyy-K-K(Dnp)-K-NH_2_ was performed and the decrease of fluorescent signal due to FRET to Dnp was monitored. The coupling efficiency data were normalized to the highest value to give the screening score. Average ligation screening score represents the sum of ligation score of the respective enzyme divided by the number of reactions screened. Top panels: heat map indicating the screening score for Ptl and Oml-1. Bottom panel: difference plot to highlight differences between Ptl and Oml-1. The improvement in screening score is given in %. Nonreactive prolines are omitted from the comparison.

In comparison to the wild-type Ptl, the novel ligase variant Oml-1 proved to be superior with a significantly broadened acyl acceptor substrate scope and increased synthetic performance (Fig. 3). Especially the acceptance of polar and small P1′ amino acids (Gln, Asn, Thr, Gly) was significantly improved, accompanied by a moderate improvement for charged P1′ residues (Glu, Arg, Lys) (Fig. 3). In addition, the acceptance of substrates bearing a residue with a branched α-carbon (Ile, Leu) at P1′ was also strongly improved (Fig. 3). Acyl acceptor peptides with a proline at P1′ or P2′ were not accepted, regardless of the flanking amino acid residue.

The substrate scope of the S2′ pocket in general was also dramatically broadened, but a mild preference for peptides with a hydrophobic amino acid in position P2′ remained (Fig. 3, top right panel). Whereas the acceptance of large hydrophobic and branched apolar residues at P2′ was clearly improved in Oml-1 as compared to the parent peptiligase, a more modest improvement was observed in the acceptance of peptides with charged or large polar P2′ residues (e.g. Asp, Glu, Arg, Lys, Gln, Asn) (Fig. 3, bottom panel - difference plot). Despite the improved coupling of substrates bearing large hydrophobic and branched apolar residues at P2′, we considered that the activities of Oml-1 remained sub-optimal when charged or polar residues at P2′ are combined with positively charged residues or large hydrophobic residues at P1′ (Fig. 3, top right panel).

Interestingly, His was well accepted at position P2′ in the acyl acceptor by Oml-1 but much less so when it was present at position P1′. Moreover, charged and polar residues at position P1′ in combination with large hydrophobic residues at P2′ were very suitable substrates. In contrast, in combination with polar and charged residues in position P2′ the synthetic yield of the peptide coupling decreased significantly (Fig. 3).

By fully mapping the acyl donor sequence space, we found the S2′ pocket to be more discriminating on substrate recognition than previously observed. Combinations of two charged residues at positions P1′ and P2′ are poorly accepted and do not represent suitable substrates (Fig. 3, top right panel). Interestingly, two substrates, the Gly-Phe and Ala-Phe variants of the acceptor Xxx-Yyy-Lys-Lys(Dnp)-Lys-NH_2_, were among the best substrates for peptiligase, but were poorly accepted by Oml-1. The peptiligase parent has a very narrow P1′ substrate scope and we reasoned that widening the S1′ pocket leads to a loss in binding affinity, thereby leaving the P2′ amino acid, i.e. Phe, as the sole driver for substrate binding. Nevertheless, Oml-1 represents a significantly improved enzyme over peptiligase, especially in terms of versatility. The average ligation screening score for nearly all 400 screened reactions was improved by 90%, with the average value increasing from 0.19 for peptiligase to 0.36 for Oml-1 (Fig. 3).

### 3.4 Crystal structures of omniligase variants

Omniligase-1 and several related mutants were subjected to crystallization to solve X-ray structures. We repeatedly observed that single point mutations in the substrate-binding region have a large effect on the crystallization of omniligase variants. Despite extensive screening of thousands of conditions, Oml-1 itself refused to form crystals. Fortunately, six closely related ligases called Pre-1 to Pre-6 [PDB: 7AM3, 7AM4, 7AM5, 7AM6, 7AM7, 7AM8] could be crystallized (Tables S2, S3), with Pre-6 differing from Oml-1 by a single point mutation (W189F). The determined crystal structures were later used in computational methods (see below).

Crystals of Pre-1 (Ptl-M222P) and Pre-2 (Ptl-M222P/L217H, the starting enzyme, see above) grew in space group P4_1_2_1_2 (no. 92), called crystal form *A,* and crystals of variant Pre-3 (Ptl-M222P/L217H/A225N) were obtained in space group P2_1_2_1_2_1_ (no. 19) or crystal form *B* (Table S3). Inspection of the crystal contacts of Pre-1, Pre-2 and Pre-3 revealed that at position F189 (S2′ pocket) the enzymes in crystal form *A* as well as in crystal form *B* have close contact (3.7 Å) with Asn109 of a symmetry-related molecule. A Trp instead of a Phe at position 189 would be too close to this Asn (2.0 Å W189-CD1 to ND1 or OD1 of N109) which cannot adopt a different conformation because of steric hindrance. Thus, Oml-1 and other omniligase variants containing mutation F189W, which is crucial for the broad substrate scope, cannot be crystallized in crystal form *A* or *B*.

Crystals of variants containing a Trp at position 189 were only found after a long incubation time for Pre-4 (Ptl-M222P/L217H/A225N/F189W) and Pre-5 (Ptl-M222P/L217H/A225N/F189W/N218D). Both crystallized in space group P4_1_22 (no. 91), called crystal form *C* (Table S3)*.* The structures of Pre-4 and Pre-5 each contain three protein molecules in the asymmetric unit (molecules A-C). In both of these variants Trp189 is involved in intermolecular contacts which are different from those in Pre-1-Pre-3. In Pre-4 and Pre-5, Trp189 of molecule A has T-stacking interactions with Tyr6 of molecule C, Trp189 of molecule B is close to Ser161 of molecule A and Trp189 of molecule C has no interactions. Mutation S218D in Pre-5 is involved in the S1′ and S2′ pockets but has no influence on the structure.

In addition to the role of Phe189 in crystal formation, we observed that for both Pre-4 and Pre-5, crystallization resulted in at least one substrate binding site occupied by a peptide sequence. Variants Pre-4 and Pre-5 crystallized in crystal form *C* containing three protein molecules (A, B and C) in the asymmetric unit. The structures of molecules A and B are highly similar with an RMSD (root-mean square deviation) of 0.3 Å. They have oxidized cysteine and an unoccupied active site like ligases Pre-1 to Pre-3. In crystal form *C* the structure of molecule C is more different with RMSDs of 0.6 – 0.7 Å to molecules A and B. It has an oxidized cysteine and a bound eglin fragment (41′-46′ (SPVTLAG P5 -P1′)) that was used as a crystallization aid (Fig. S3, S4). In this crystal form *C*, we also observed a crystallization artifact; part of the loop from Ile205 to His217 of molecule C is forcibly drawn into the active site of molecule B, mimicking a peptide substrate (Fig. S5). In the pulled loop the residues Asn212 and Lys213 have double conformations while Tyr214 has a triple conformation (Fig. S6) showing main chain mobility. Then via Leu209, Thr208, Ser207 and Cys206 the loop leaves the active site of molecule B and returns to the configuration observed in chain A and B at Ile205. The phi angle of Cys206 of chain C which has a disulfide bond with Cys3, has shifted by 20° compared to the A-molecule. Hence, the conformation of the five N-terminal residues of chain C (AKCVG) also changed. This is an example of a crystallization artifact, and shows that ligases have a strong preference for binding a peptide in the active site even if it is their sibling. Although the active site of omniligase molecule C is close to the cleavage site it does not seem to be disrupted as an intact eglin fragment is bound.

The crystal of Pre-6, that differs from Oml-1 by the single point mutation W189F, was grown in crystal form *B*, identical to Pre-3, with the same crystallization agent, polyacrylic acid (Table S3). Among other single point mutations in the omniligase variants (Table S2) only I107V effects the substrate binding pockets and reaction rates by enlarging the hydrophobic S4 subsite with one methyl group.

The different crystal forms show the flexibility of the active site of peptiligases. For instance, His217 is present in two distinct conformations, one pointing into the active site while in the other conformation the sidechain of His217 is facing the solvent. Also the sidechain of His64 is observed in two conformations. These different conformational states, showing the flexibility of the active site, are required for enzyme activity.

All conditions giving crystals in this study were employed for crystallization attempts of Oml-1 but without success. Therefore, an omniligase-1 model was constructed by superimposing the sidechain W189 of crystal form *C* (Pre-4 and Pre-5) on Pre-6. This model was used for later studies (see below).

### 3.5 The effect of the A225N mutation

**Fig. 4:**
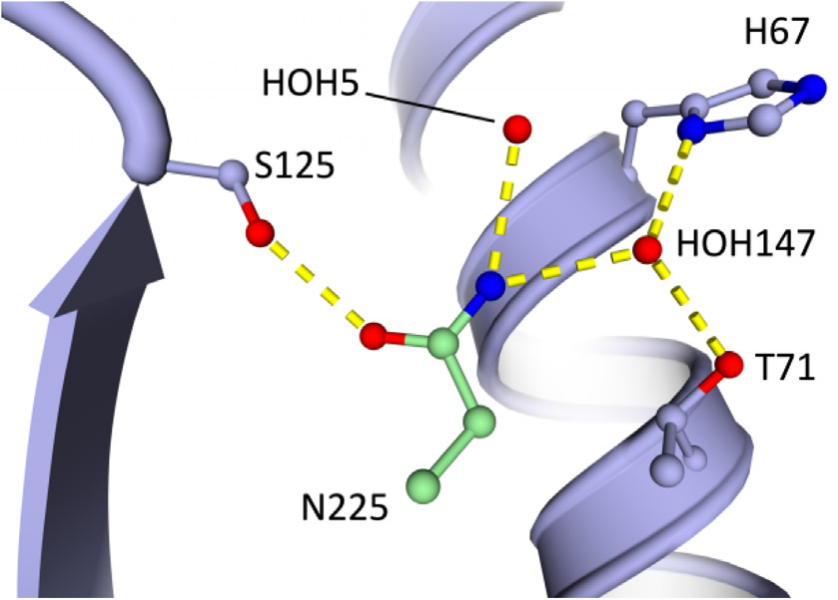
H-bonding contacts introduced by mutation A225N. For clarity, the α-helix that Asn225 itself belongs to is not shown. The illustration is based upon the Pre-6 structure (7AM8).

**Fig. 5:**
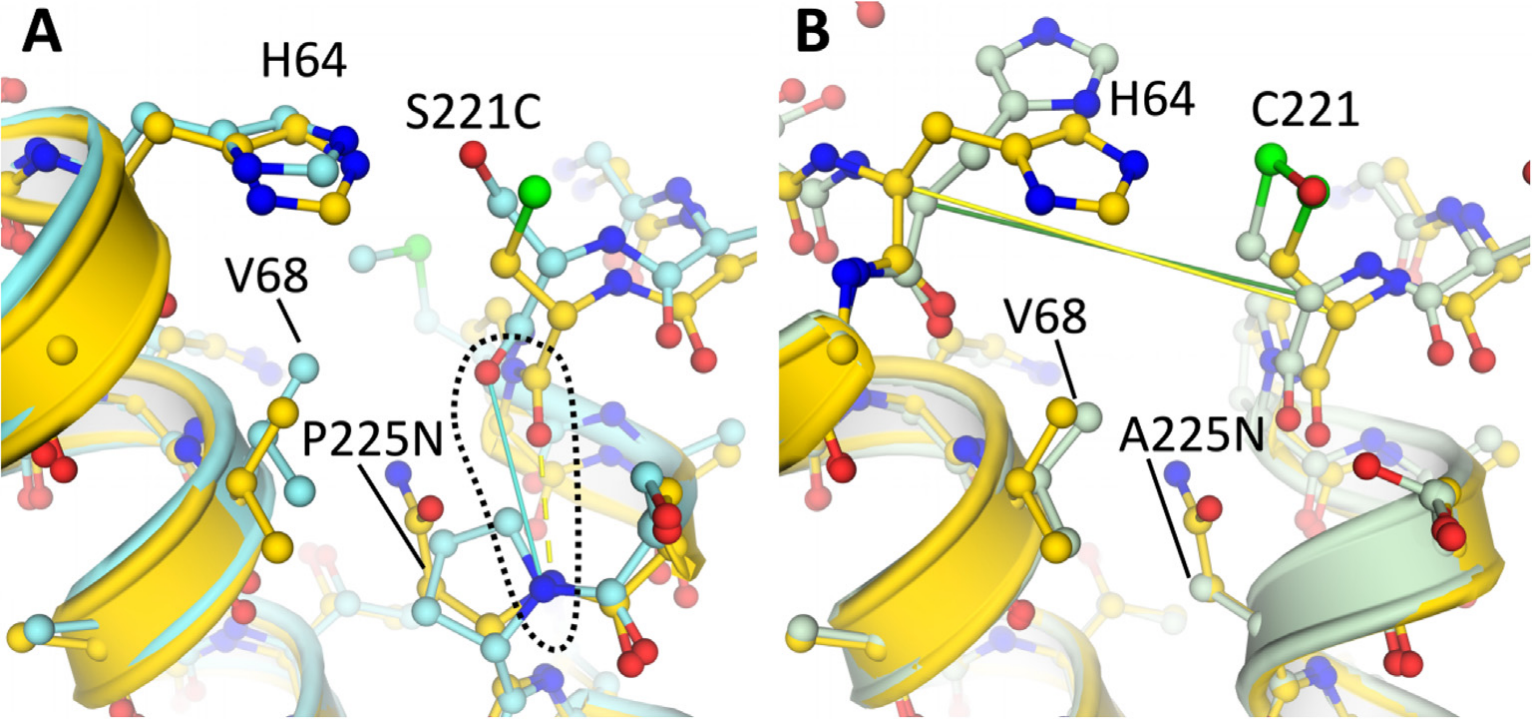
Effect of position 225 substitutions on the backbone structure. A) wild type subtilisin (1SBN, turquoise) compared to Pre-3 (yellow, with A225N mutation). The P225N mutation shortens the α-helix by allowing for backbone H-bonding between Cys221 and Asn225 (yellow dashes in panel A, blue line indicates the distances between the involved atoms in the subtilisin structure). B) Pre-2 (green) compared to Pre-3 (yellow, A225N). The straight lines indicate the distance between the Cα backbone atoms, showing that the A225N mutation pushes the helices with the catalytically active C221 and H64 residues apart. For clarity, the secondary structure is not shown when backbone atoms are relevant. C221 in the Pre-2 X-ray structure was oxidized to cysteine sulfenic acid. (For interpretation of the references to colour in this figure legend, the reader is referred to the web version of this article.)

In comparison to the Pre-1 and Pre-2 variants, Pre-3 has the additional mutation A225N at the S1′ pocket that is also present in Oml-1. This remarkable A225N mutation almost doubles the S/H ratio (see above), which asks for a structural explanation. The sidechain of Asn225 in the α-helix close to the active site has a hydrogen bond to the hydroxyl group of Ser125 providing a more restrained position than in Pre-1 or Pre-2 (Fig. 4). In the latter variants, there is 50% occupancy for the sidechain of Ser125 which makes a H-bond to the catalytic triad residue Asp32, but not in any of the X-ray structures with Asn225. Furthermore, Asn225 has H-bonds to the sidechain of Asn123 and the carbonyl oxygen of the non-oxidized Cys221, and thus makes good contacts, possibly rigidifying, with the local active site environment of omniligase-1 (Fig. 4, Fig. 5). The S1′ pocket becomes shallower by ca. 2.5 Å but about 1.3 Å wider compared to variants Pre-1 and Pre-2, providing even less steric crowding (see above). A Gln at this position 225 would be too large for optimal hydrogen bonding and would shrink the S1′ pocket too much, confirming the experimental data (Table 1).

Further structural comparisons were made to understand how Asn225 could give higher S/H ratios than an alanine at that position. It was reported twice [13], [50], with results obtained from both rational design and directed evolution, that a very small residue (Gly or Ala) is required at position 225 to give good S/H ratios. The proposed mechanism is that a small residue creates space for the nucleophilic Cys221, which itself is bulkier than the original serine residue (the sulfur has a 0.3 Å larger van der Waals radius than oxygen). Inspection of the structures obtained here shows that both a P225A and a P225N substitution shorten the α-helix that ends at Cys221 because Pro225 is no longer preventing close contacts between the amide at position 225 and the carbonyl oxygen at position 221 (Fig. 5A). As a result, Cys221 is shifted by ≅ 0.5 Å, creating extra space on the side of the substrate. This observation agrees with that reported for the P225A mutation by Abrahamsen *et al.* [13], who did not deposit an X-ray structure. The asparagine side chain is larger than that of a proline, but points in a different direction; it is located between the α-helices that the catalytic triad residues Cys221 and His64 are positioned upon and as a result these residues are pushed away from each other (Fig. 5B). The Cα-Cα distances increase by 0.4 Å for residue 225 and Val68 and by approximately 1 Å for Cys221 and His64 (Table S5). The latter is in agreement with the wider S1′ binding pocket. This repositioning of the active site residues could affect the S/H ratio by influencing how a hydrolytic water or an acyl acceptor would be positioned between Cys221 and His64 just prior to nucleophilic attack.

Two modeling experiments were carried out to confirm that the introduction of Asn225 pushes nearby backbones aside, as is suggested by the slight increases in distances in the X-ray structures. The effect of all possible mutations on folding stability (ΔΔG^fold^) was modeled both using the Pre-2 X-ray structure, that carries an Ala at position 225, and the Pre-3 structure, which features Asn225. The effect of the mutations was predicted with both FoldX and Rosetta software, which gave very similar results (Table S6). For both structures, the most stabilizing residue was predicted to be an alanine while an Asn was predicted to be destabilizing by 37 kJ/mol in case of the Pre-2 structure and by 10 kJ/mol for the structure that had already an Asn present. In case of an Ala225-containing template, the energetic penalties of introducing a group larger than Ser at position 225 was much higher than when introducing a more bulky group in an Asn225-contaning template. This confirmed that introducing Asn225 created more space in between the local backbones.

In the same structures, we searched for strain as described in Section 2.5. Using the Amber ff14SB force field, the relative van der Waals energy (Lennard-Jones) of each non-hydrogen atom was mapped on protein structures in which the packing of Asn225, and its surroundings, had been optimized using FoldX (Fig. S8). In an Ala225 template (Pre-2) clearly more strain was visible on both Val68 and Asn225 than when examining the Asn225 template (Pre-3). The occurrence of some strain even in the Asn225 template explains that FoldX and Rosetta_ddg predictions suggest that the N225A mutation should be stabilizing (Table S6). However, FoldX and Rosetta calculations do not include explicit water molecules, which may cause inaccurate results for the A225N substitution since it makes good contacts with water (Fig. 4).

In summary, the A225N mutation could improve the S/H ratio by shortening the Cys221 helix in the same way as the P225A mutation, creating space for the cysteine. The A225N substitution also widens the S1′ binding pocket by pushing His64 and Cys221 apart, which is facilitated by formation of H-bonds to nearby residues and to two water molecules in the S1′ pocket.

### 3.6 Modeling omniligase selectivity

**Fig. 6:**
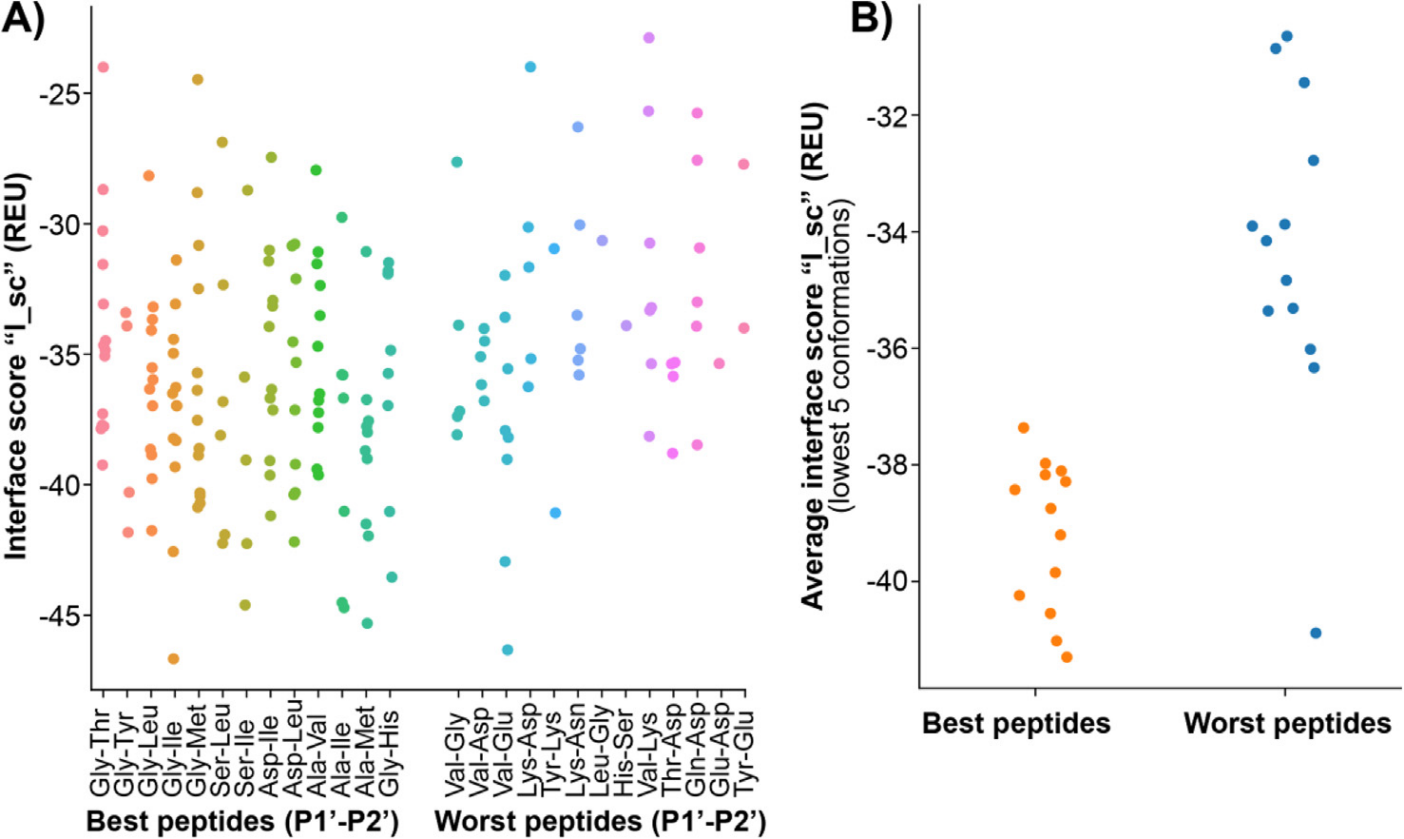
Rosetta FlexPepDock interface score “I_sc” values for the ligands modeled as the respective products DFSKL-P1’-P2’-K. A) Interface scores “I_sc” for all ligand conformations that passed the geometric criteria for productive binding modes. B) Average interface score “I_sc” for the lowest 5 conformations per ligand.

The experimental screening of Oml-1 with a library of nucleophilic peptides demonstrated an impressive improvement of the substrate range. Computational methods can assist enzyme engineering by providing a way to explore a larger sequence space in a more efficient and less laborious manner compared to laboratory evaluation. To examine if ligand affinity can explain Oml-1 selectivity and if the relationship between affinity and activity can be predicted computationally, we examined a subset of 26 peptides from the substrate library, corresponding to the 13 best and 13 worst ligands (Table S7). Using crystal structures of subtilisins in complex with peptide ligands, we generated the expected backbone conformation for peptide ligands, onto which the side chains of the best and worst ligands were modeled as the corresponding peptide product. To this end, the starting conformation from Pre-6 (PDB: 7AM8) and Rosetta were used to model the desired enzyme-ligand complexes. Next, from these complexes, conformational diversity was generated using the Rosetta backrub protocol and the conformations were further refined using the Rosetta FlexPepDock high-resolution minimization protocol [45]. Finally, generated conformations of productive binding modes were ranked using the average interface score “I_sc” obtained with FlexPepDock (Fig. 6). The results show a considerable spread of interface score values obtained within the geometric constraints that define the productive binding modes (Fig. S9). This is more evident for the best peptides, as more productive binding conformations were generated than with peptides that were not good ligands. Therefore, to rank the peptides by their reactivity as a nucleophile, we used interface score values averaged over up to 5 of the lowest-energy conformations (see Section 2.5, Fig. 6).

The observation that the interface score “I_sc” can distinguish the best 13 ligands from the 13 worst ligands (Fig. 6) suggests that the selectivity of Oml-1 in this dataset is mainly determined by the binding energy of the P1′ and P2′ amino acids. Therefore, we used the Rosetta-generated binding mode predictions for the 26 different ligands to gain a better understanding of the selectivity preferences of Oml-1.

**Fig. 7:**
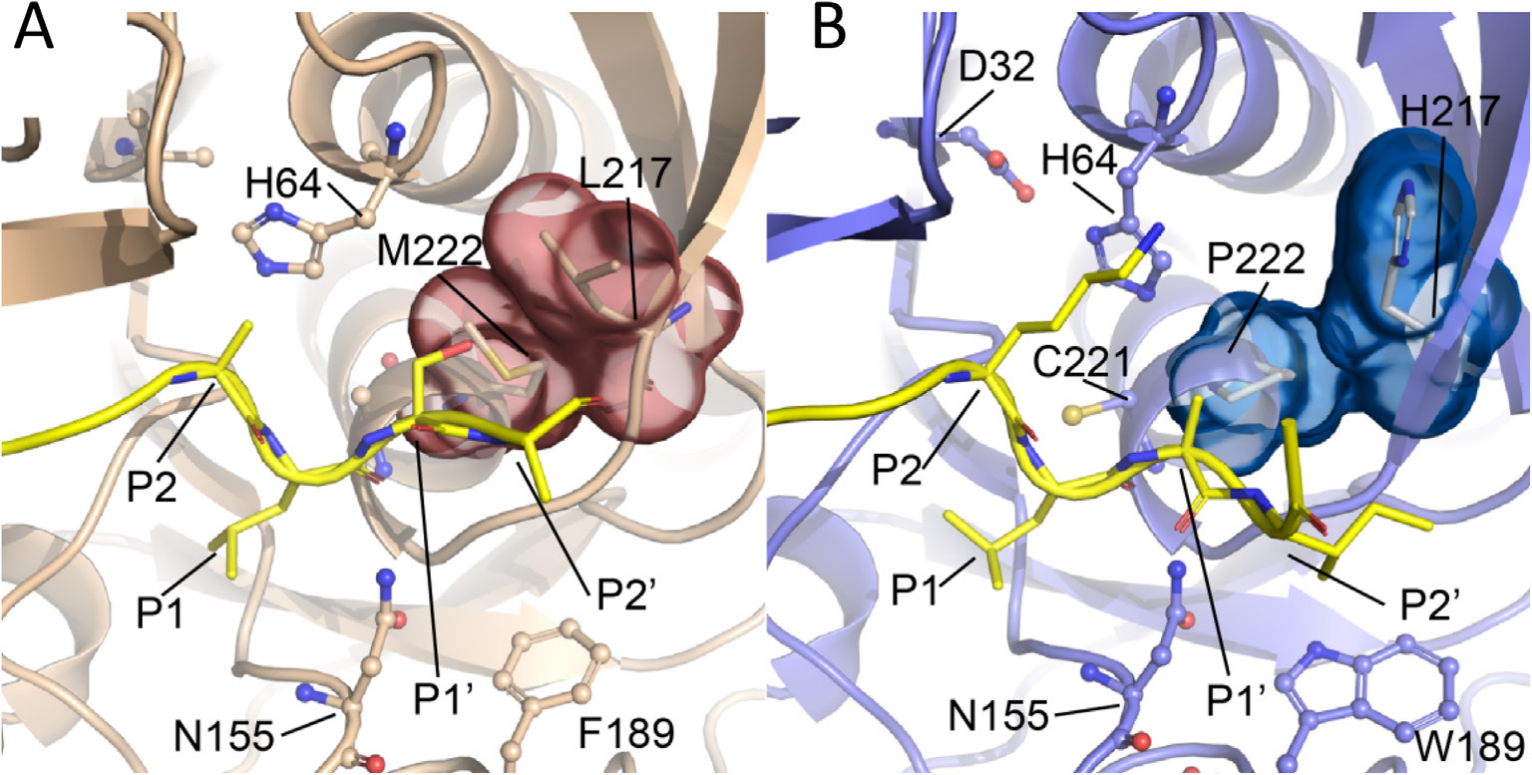
Effect of the L217H-M222P mutation on the S1′ cavity. A) the wild-type amino acids M222-L217 shown in subtilisin (PDB 3BG0). B) model of the binding mode of the peptide DFSKL-P1′(A)-P2′(I)-K bound to Oml-1. The peptide ligands are shown in yellow and the mutated amino acids highlighted with a surface representation. (For interpretation of the references to colour in this figure legend, the reader is referred to the web version of this article.)

The 13 best ligands docked in Oml-1 contain smaller amino acids such as Ala, Gly, Ser or Asp at position P1′ (Table S7, Fig. 3). In contrast, the docked 13 worst ligands possess large polar residues at P1′, such as Thr, Gln, Glu, Lys and His, or large hydrophobic residues, such as Val, Leu and Tyr. Analysis of the Rosetta binding mode predictions showed that small side chains at P1′ bind between the Lys side chain of the peptide at P2 and residues His67, His217 and Pro222 that form the S1′ cavity, whereas larger groups at P1′ are either exposed to the solvent or show steric clashes in the S1′ cavity or the side chain of P2-Lys. In the peptiligase parent, the hydrophobic interaction between Leu217 and Met222 limits the space available for a buried P1′ side chain in the S1′ cavity. On the other hand, His217 introduced in Oml-1 can rotate away and become exposed to the solvent, enlarging the S1′ cavity and allowing slightly larger P1′ side chains to bind. This effect is particularly noticeable for P1′ amino acids that can form favorable interactions with Pro222, such as Ala, Thr and Met (Fig. 3, Fig. 7). In conclusion, the L217H + M222P mutations in Oml-1 increase the P1′ tolerance due to an enlarged S1′ cavity that arises when His217 rotates towards the solvent. However, the tolerance for bulky P1′ amino acids is still limited due to the large side chain of the lysine that is present at P2 in all substrates.

At the P2′ position of the ligands, Oml-1 prefers large hydrophobic residues, while polar residues, in particular charged residues, are disfavored (Fig. 3). The 13 best docked peptide ligands contain mostly large hydrophobic residues at P2′, such as Val, Ile, Leu, Met, Phe or Tyr, while the worst 13 ligands contain polar residues Ser, Asn, Asp, Glu and Lys or Gly (Table S7). An analysis of the docked poses showed that the large hydrophobic residues at P2′ in the best ligands were shielded from the solvent through hydrophobic interactions with Trp189. The polar residues at P2′ of the poor peptide ligands were either exposed to solvent or hydrogen bonded to the active site Asn155, displacing it from an orientation where it can contribute to the oxyanion hole. Rosetta output structures showed that Trp189 introduced in Oml-1 by mutation F189W should point to the hydrophobic cavity formed by Ala179, Ala187, Pro188, Gly202, Val203, Gly219 and Thr220 which is occupied by Phe189 in peptiligase. Due to its larger size Trp189 cannot become as shielded from the solvent as Phe189, and the additional hydrophobic interactions offered by the indole ring can serve as a driving force to bind large hydrophobic residues at P2′. Strong hydrophobic interactions between P2′ amino acids and Trp189 can even overcome the presence of bulky or polar residues at P1′ that would compromise peptide binding due to a poor accommodation in the S1′ pocket.

### 3.7 Omniligase-1 in practical synthesis

**Fig. 8:**
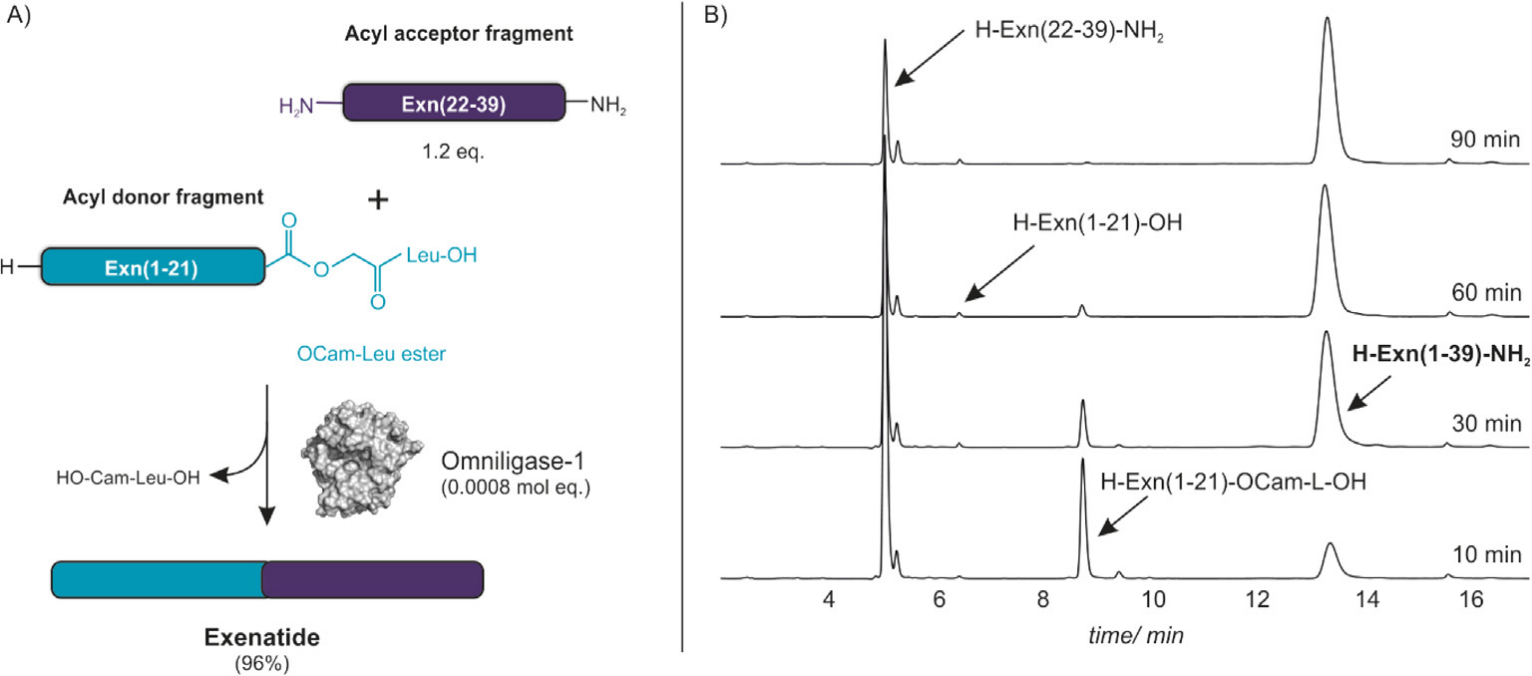
Omniligase-1 catalyzed ligation of Exn(1–21)-OCam-L-OH with Exn(22–39)–NH_2_. A) Reaction scheme; B) HPLC chromatograms of the ligation reaction. HPLC profiles were determined after the ligation has proceeded for 10, 30, 60 and 90 min. After 90 min full consumption of Exn(1–21)-OCam-L-OH was observed with formation of Exn(1–39)–NH_2_ in 96% yield and 4% hydrolysis.

To illustrate the applicability of Oml-1 we used the enzyme in the synthesis of an important pharmaceutical peptide. Exenatide is a 39 amino acid peptide with antihyperglycemic activity [51]. It can be enzymatically synthesized in coupling reactions starting from two chemically synthesized fragments: Exn(1–21)-OCam-L-OH (HGEGTFTSDLSKQMEEE**AVRL**-OCam-L-OH) and Exn(22–39)-NH_2_ (**FI**EWLKNGGPSSGAPPPS-NH_2_) (Fig. 8). When performing these coupling reactions, only very small amounts of Oml-1, i.e. 0.0008 molar equivalents compared to the acyl donor fragment, were needed to catalyze the efficient synthesis of the 39-mer exenatide. The yield clearly improved by 10% as compared to previously described ligation reactions [9]. Furthermore, hydrolysis of activated acyl donor to side product amounted to only 4%. The use of Oml-1 in CEPS of exenatide was also demonstrated at 100 g scale in a sustainable and cost-efficient process [28].

A possible side reaction during kinetically controlled enzymatic peptide bond synthesis is cleavage of the acyl-enzyme intermediate by an unprotected N-terminus of the acyl donor instead of by acyl acceptor, which would give useless side product by dimerization of the donor. This was not observed in exenatide synthesis reactions catalyzed by Oml-1. The lack of side product formation is in accordance with much better ligation score of Oml-1 for the acyl acceptor P1′-P2′ sequence Phe-Leu (ligation score 0.5) over the His-Gly sequence of the acyl donor (0.1). This selectivity of the S1′ and S2′ pockets thus allows exenatide synthesis without the need for *N*-terminal protection of the activated acyl donor fragment (Fig. 8).

## 4 Conclusions

The introduction of a broad substrate range into peptiligase to obtain the derivative omniligase-1 as reported here represents a considerable extension of the available set of ligases and a clear improvement in comparison to the parent peptiligase. For the second generation peptiligase variants we identified S1′ and S2′ pockets residues that play an important role in the recognition of the acyl acceptor substrate. The creation of site-saturation libraries of position A225 (S1′ pocket) and F189 (S2′ pocket) was crucial to expediently identify improved peptiligase variants with a broadened substrate scope and/or an increased synthetic performance. Two key mutations were found, A225N and F189W, and were successfully combined with the reaction rate-improving mutation I107V, resulting in the clearly improved peptiligase variant omniligase-1.

Subsequent determination of the X-ray crystal structures of Oml-1 precursors and creation of computational models of peptide binding modes in Oml-1 suggest that the broadened substrate scope is a combination of two effects. The S1′ pocket mutations L217H + M222P increase slightly the P1′ tolerance due to an enlarged S1′ cavity allowed by the elimination of the hydrophobic patch and ability to make His217 exposed to the solvent. The second effect is due to F189W in the S2′ pocket, which serves as a strong binding force to drive the binding of substrates with hydrophobic residues at P2′.

The entire combinatorial substrate scope for positions P1′ and P2′ was mapped for Oml-1 and revealed that even under suboptimal (screening) reaction conditions omniligase-1 can accept many of the 400 possible acyl acceptor substrates. As demonstrated for exenatide, after mapping, it is even possible to exploit differences between accepted P1'-P2' combinations for distinguishing N-termini of an acyl donor and an acyl acceptor, and thereby design CEPS where acyl donor N-terminal protection is obsolete. From an application point of view, these results confirm the broad applicability of omniligase-1 in chemo-enzymatic peptide synthesis.

## Declaration of Competing Interest

AT, TN, RdV, LKMM are employees of EnzyPep and some of EnzyPep’s products are (enzymatically synthesized) peptides.
