# Supplementary material for: From thiol-subtilisin to omniligase: Design and structure of a broadly applicable peptide ligase
Source: Comput Struct Biotechnol J. 2021 Feb 9;19:1277–87. doi: 10.1016/j.csbj.2021.02.002 (PMC7921005; doi:10.1016/j.csbj.2021.02.002)
Supplement: Supplementary data 1 [file mmc1.pdf]

From Thiol-Subtilisin to Omniligase: Design and Structure of a Broadly Applicable Peptide Ligase

Supplementary Information

Ana Toplak,[a] Eduardo F. Teixeira de Oliveira,[b] Marcel Schmidt,[a] Henriëtte J. Rozeboom,[b] Hein J. Wijma,[b] Linda K.M. Meekels, Rowin de Visser, [a] Dick B. Janssen,[b#] and Timo Nuijens [a#]

[a]EnzyPep B.V., Brightlands Campus Urmonderbaan 22, 6167 RD Geleen (The Netherlands)
[b]Biotransformation and Biocatalysis, Groningen Biomolecular Sciences and Biotechnology Institute,

University of Groningen Nijenborgh 4, 9747 AG Groningen (The Netherlands)
# Corresponding authors:

Dick B. Janssen: d.b.janssen@rug.nl
Timo Nuijens: timo@enzypep.com

Solid Phase Synthesis of the Peptide Substrates

Solid phase peptide synthesis (SPPS) of acyl donors for screening (*i.e.* Abz-KFTKL-Cam-L-OH or alternatively Ac-DFSKL-Cam-L-OH) was carried out using standard protocols for Fmoc deprotection using 20% (v/v) piperidine in dimethylformamide (DMF; 2x 8 min) and coupling of the amino acids (4 eq.) with diisopropylcarbodiimide (DIC; 4 eq.) and OxymaPure (4 eq.) for 2x 20 min with 10 mL of solvent per g of resin were used standardly. The Cam-ester was introduced using Fmoc-glycolic acid according to Nuijens *et al.*[1]

The fluorescent group Abz was introduced as Boc-2-Abz-OH using standard SPPS procedures and in the case of Ac-DFSKL-Cam-L-OH acetylation was performed using a solution of 0.5/0.5/90 (v/v/v) acetic anhydride/N,N-diisopropylethylamine (DIPEA)/DMF for 30 min. Final peptide cleavage from the resin was performed using a mixture of 95/5/2.5 (v/v/v) TFA/triisopropylsilane (TIS)/water for 120 min before the peptide was precipitated in cold diisopropylether (10 mL TFA cleavage mixture per 90 mL solvent). After washing the crude peptide three times it was dried *in vacuo* and analyzed by HPLC-MS. Finally, peptides were purified by preparative HPLC and lyophilized. For acyl acceptor synthesis Fmoc-Lys(Boc)-Wang resin was swollen in DMF (2x 5 min), before Fmoc deprotection was performed with 20% (v/v) piperidine in DMF (2x 8 min) and by washing with DMF (6x 2 min). Fmoc-Lys(Dnp) (1.2 eq.; Dnp= 2,4-dinitrophenyl) was coupled with 1.2 eq. DIC and 1.2 eq. OxymaPure in DMF (2x 20 min). After Fmoc deprotection and washing the residual standard Fmoc-amino acids were coupled (4 eq.) using 4 eq. DIC and 4 eq. OxymaPure for 40 min in DMF. After 20 min again 4 eq. of DIC were added. Peptide cleavage from the resin was performed using a 95/5/2.5 (v/v/v) mixture of TFA/TIS/water for 120 min before the peptide was precipitated in cold diisopropylether (10 mL TFA cleavage mixture per 90 mL solvent). After washing the crude peptide three times it was dried *in vacuo* and analyzed by HPLC-MS. In order to create a library of 400

different acyl acceptor fragments H-Xxx-Yyy-K-K(Dnp)-K-OH all respective combinations of 20 proteinogenic amino acids were coupled in positions P1’ (Xxx) and P2’ (Yyy).

The peptide purity was assessed using an Agilent 1260 Infinity HPLC system coupled with an Agilent 6130 quadrupole mass spectrometer (Agilent, Santa Clara, CA, USA) to determine the peptide mass. Separation was performed using Dr. Maisch ReProSil-Pur C18 5μm, 4.6 x 250 mm or Waters XSelect CSH C18, 2.5 μm particle size, 150× 3.0 mm column, eluting with 0.05% (v/v) MSA in a water acetonitrile gradient, with a flow rate of 1 mL/ min. As mobile phase a binary mixture of A (water + 0.05% (v/v) MSA) and B (acetonitrile (ACN) + 0.05% (v/v) MSA) was used. A linear gradient from 5 to 98% B in 21 min, followed by isocratic 95% B for 5 min was used by default. For optimal results an appropriate gradient was chosen for each sample individually. The purity of peptides was determined by automatically integrating product and impurity peaks of the relevant HPLC spectrum (λ= 220 nm).

Computational Protocols

Peptide modeling

To create an initial guess on how the peptides bind to omniligase-1 we collected and superimposed X-ray structures of subtilisins with peptides bound in the binding cleft (1CSE, 1LW6, 1OYV, 1R0R, 1SBN, 1SIB, 1SPB, 1TM1, 1TM3, 1TM4, 1TM5, 1TM7, 1TMG, 1TO1, 1TO2, 1V5I, 1Y1K, 1Y33, 1Y34, 1Y3B, 1Y3C, 1Y3D, 1Y3F, 1Y48, 1Y4A, 1Y4D, 1YU6, 2SEC, 2SIC, 2SNI, 3BGO, 3CNQ, 3CO0, 3SIC, 5OX2, 5SIC). The backbones of these peptides show low conformational variability between P4 and P2’ amino acid positions. Therefore, we used the backbones of the superimposed X-ray structures to obtain the average backbone position from P5 to P3’. The position of the average backbone is identical to the one found in the X-ray structure 1R0R, with and RMSD for atoms “N”, “C” and “CA” of 0.3 Å.

The P5 to P3’ backbone in the average position was combined with the structure Pre-6 [PDB: 7AM8] and the desired peptide and omniligase-1 complex generated during the backrub protocol.

“Backrub” protocol

To sample the local conformational space of the protein-peptide complex we adapted the Rosetta Scripts XML protocol from Barlow *et al*.[3]

One of the adaptations was to use 4 “AtomPair” constraints to keep the active site residues Asp32, His64, Asn155, Cys221 and the p1 amino acid close to the crystal structure. Therefore we used “GAUSSIANFUNC” constraints between the pair His64 “HD1” atom and Asp32 “OD1” atom to maintain the hydrogen bond, between the pair Cys221 “HG” atom and His64 “NE2” atom to maintain the hydrogen bond, between the pairs Cys221 “H” atom and p1 “O” atom and between Asn155 “1HD2” atom and p1 “O” atom to maintain the oxyanion hole.

During the “backrub” protocol the number of minimizations was limited to 2500 steps with the default harmonic restraints on the Cα pairs of atoms within 9 Å of each other. To define the residues in the mutation neighborhood we used the default distance of a Cβ atom (Cα for glycines) within 8 Å of any mutant position. “Backrub” was run at a temperature of 1.2 kT, for up to 15,000

backrub Monte Carlo trials/steps. Approximately 150 output models were generated for each substrate.

"FlexPepDock" protocol

The binding mode conformations generated by the "backrub" protocol were further minimized and ranked using the FlexPepDock high-resolution minimization protocol.

The following flags were used:

-flexPepDocking:receptor_chain A
-flexPepDocking:peptide_chain B
-flexPepDocking:abinitio False
-flexPepDocking:lowres_preoptimize False
-flexPepDocking:pep_refine False
-flexPepDocking:flexPepDockingMinimizeOnly True
-packing::use_input_sc True
-packing::ex1:level 4
-packing::ex2:level 4
-packing::ex2aro:level 4

Selection of productive binding modes

The visual inspection of the conformations after minimization with "FlexPepDock" revealed that many of the generated binding modes displace His64, Asn155 or the P1 amino acid from the active site to allow accommodation of the different peptide side chains. As a result, "FlexPepDock" energy terms are evaluated on non-productive binding modes of the peptide. To discard these non-productive binding modes, three geometric criteria were implemented (Fig. S9).

Supplementary Tables

Table S1: Synthetic performance of Pro225 mutants. The enzymes were tested in synthesis reactions using 3.3 mM nucleophile (H-SLR-NH_2) and 8.3 mM acyl donor (Ac-DFSKL-OCam) in 0.08 M phosphate buffer pH 8.0 with 1 ml total volume. To the reaction mixture 5.5 ug of enzyme was added and incubated for 30 min with shaking at room temperature. The reaction was stopped by quenching with 1%v/v MSA in water to a final 1:3 volume ratio and analyzed on LC-MS. The synthetic product, the hydrolysed acyl donor, and the remaining pentapeptide C-terminal Cam-ester were quantified from peak areas.

**Pro225X**	**Synthesis (%)**	**Hydrolysis (%)**	**S/H ratio**
Asn	88	12	7.3
Asp	87	13	6.7
Ser	85	14	6.1
Cys	84	16	5.3
Val	81	19	4.3
Ile	80	20	4.0
Leu	78	22	3.5
Thr	77	18	4.3
Gly	74	16	4.6
Ala	67	15	4.5
His	46	25	1.8
Gln	42	29	1.4
Phe	22	31	0.7
Glu	9	25	0.4
Pro	6	35	0.2
Lys	2	30	0.1
Tyr	2	60	0.0
Met	1	32	0.0
Arg	1	45	0.0
Trp	1	67	0.0

Table S2. Mutations in omniligase and other peptiligase variants.

interaction site	position	peptiligase/omniligase variant								
		OL1	Pre-6	Pre-5	Pre-4	Pre-3	Pre-2	Pre-1	Thy 5OX2	Ptl
backbone	9	A	A	S	S	S	S	S	A	A
backbone	31	L	L	I	I	I	I	I	L	L
S4	107	V	V	I	I	I	I	I	I	I
	156	S	S	S	S	S	S	S	N	S
	166	S	S	S	S	S	S	S	D	S
S2	189	W	F	W	W	F	F	F	W	F
backbone	212	G	G	N	N	N	N	N	G	G
S1’	217	H	H	H	H	H	H	L	R	L
S2’	218	S	S	D	S	S	S	S	S	S
S1’	222	P	P	P	P	P	P	P	G	M
S1’	225	N	N	N	N	N	A	A	N	A

Table S3. Crystallographic data and refinement statistics. Values in parentheses are for the highest resolution shell.

**Data collection**	Pre-1	Pre-2	Pre-3	Pre-4	Pre-5	Pre-6
Crystal Form	*A*	*A*	*B*	*C*	*C*	*B*
Space group	$P4_12_12$ (92)	$P4_12_12$ (92)	$P2_12_12_1$ (19)	$P4_122$ (91)	$P4_122$ (91)	$P2_12_12_1$ (19)
Unit cell (Å), a, b, c	58.5, 58.5, 126.0	58.4, 58.4, 125.3	53.7, 60.0, 78.4	105.4, 105.4, 192.1	105.8, 105.8, 191.5	54.5, 60.7, 78.9
Molecules per A.U.	1	1	1	3	3	1
V_M ($Å^3$ Da^{-1}) [2]	1.8	1.8	2.2	3.0	3.0	2.2
Resolution range (Å)	42.9 - 1.61 (1.64 – 1.61)	42.7 - 1.81 (1.85 – 1.81)	44.4 – 2.3 (2.38- 2.30)	64.0 -2. 70 (2.83 -2.70)	48.6 - 2.61 (2.72 – 2.61)	48.1 – 2.04 (2.09 - 2.04)
N° total measurements	220804 (8660)	156418 (7644)	45568 (4199)	161792 (20167)	270897 (30352)	108182 (6918)
N° unique reflections	29222 (1315)	20564 (1127)	11252 (1083)	29451(3170)	33957 (4033)	17317 (1217)
R_{merge} (%)	4.7 (18.4)	5.4 (26.5)	20.0 (53.7)	26.6 (104.8)	24.7 (91.7)	15.0 (69.0)
R_{pim} (%)	1.9 (8.1)	2.2 (11.8)	10.5 (33.0)	12.2 (49.4)	9.9 (38.1)	7.0 (34.9)
Completeness (%)	99.6 (91.6)	99.7 (94.9)	96.6 (96.6)	96.8 (93.9)	99.8 (98.8)	99.5 (97.2)
Average I/σI	26.0 (8.9)	23.8 (6.4)	3.7 (1.5)	6.1 (1.5)	8.3 (2.2)	9.0 (2.4)
Multiplicity	7.6 (6.6)	7.6 (6.8)	4.0 (3.9)	5.5 (5.4)	8.0 (7.5)	6.2 (5.7)
$CC_{1/2}$	0.999 (0.982)	0.999 (0.959)	0.973 (0.802)	0.969 (0.369)	0.990 (0.583)	0.995 (0.593)
**Refinement**						
Contents of A.U.						
Protein	1 – 278	1 – 278	2 – 278	A: 1-276, B: 1-278, C: 1-278	A: 1-276, B: 1-277, C: 1-275	2 – 278
waters	320	233	190	45	151	232
other	2 sulfate ions, 4 glycerol molecules	3 sulfate ions, 3 glycerol molecules	2 sodium ions	2 tartrate ions, 8 glycerol molecules	5 sulfate ions, 9 glycerol molecules, 1 PEG molecule	1 prop-2-enoic acid (acrylic acid), 1 chloride, 2 sodium ions
Inhibitor				Eglin residues 40-46	Eglin residues 41-46	
R/ R_{free}	13.4 / 15.9	13.6 / 17.2	23.0 / 28.7	21.2 / 25.4	19.5 / 23.7	18.1 / 22.8
**Geometry**						
r.m.s.d. bonds (Å)	0.010	0.010	0.004	0.010	0.009	0.009
r.m.s.d. angles (°)	1.6	1.6	1.3	1.7	1.5	1.5
**PDB accession code**	7AM3	7AM4	7AM5	7AM6	7AM7	7AM8

Table S4. Root-mean square deviations (RMSDs) in Å of 266 aligned residues of the variants of omniligase-1

	Pre-1	Pre-2	Pre-3	Pre-4A	Pre-4B	Pre-4C	Pre-5A	Pre-5B	Pre-5C	Pre-6
Pre-1	-	-	-	-	-	-	-	-	-	-
Pre-2	0.16	-	-	-	-	-	-	-	-	-
Pre-3	0.57	0.59	-	-	-	-	-	-	-	-
Pre-4A	0.56	0.57	0.50	-	-	-	-	-	-	-
Pre-4B	0.59	0.60	0.44	0.25	-	-	-	-	-	-
Pre-4C	0.83	0.83	0.70	0.66	0.62	-	-	-	-	-
Pre-5A	0.54	0.56	0.50	0.21	0.29	0.67	-	-	-	-
Pre-5B	0.63	0.65	0.45	0.32	0.19	0.62	0.27	-	-	-
Pre-5C	0.87	0.97	0.74	0.71	0.69	0.18	0.70	0.65	-	-
Pre-6	0.57	0.59	0.32	0.46	0.42	0.68	0.45	0.43	0.71	-

Table S5. Effect of the A225N substitution on helix to helix distances in structures of subtiligase derived variants.

variant	PDB code	residue at position 225	Cα-Cα distance between Cys221 and His64 (Å)	Cα-Cα distance between residue 225 and Val68 (Å)
Pre-1	7AM3	Ala	7.7	5.4
Pre-2	7AM4	Ala	7.7	5.4
Pre-3	7AM5	Asn	8.6	5.7
Pre-4[A]	7AM6	Asn	8.8 / 9.0 / 9.3	5.9 / 5.8 / 6.1
Pre-5[A]	7AM7	Asn	8.9 / 9.0 / 9.4	6.0 / 5.9 / 6.2
OL-W189F	7AM8	Asn	8.6	5.8
Thymoligase	5OX2	Asn	9.0	5.8

[A] three monomers in the unit cell.

Table S6. Strain relief by backbone movements demonstrated by stability change predictions.

computational protocol	with the A225 template (kJ/mol) [A]			with the N225 template (kJ/mol) [A]		
	FoldX	Rosetta 3	Rosetta 6	FoldX	Rosetta 3	Rosetta 6
**A225A**	**0**	**0**	**0**	**0**	**0**	**0**
A225C	18	18	18	3	12	13
A225D	47	42	42	12	23	23
A225E	53	55	55	10	24	25
A225F	140	91	89	63	55	55
A225G	5	14	13	3	14	14
A225H	108	80	79	55	37	38
A225I	43	53	53	20	31	32
A225K	56	77	77	20	55	55
A225L	45	61	61	18	32	31
A225M	40	37	36	7	19	20
**A225N**	**36**	**38**	**38**	**7**	**13**	**13**
A225P	19	24	25	18	26	26
A225Q	54	52	52	12	14	15
A225R	70	87	86	34	60	61
A225S	8	14	14	8	8	8
A225T	24	32	32	12	19	19
A225V	29	35	35	15	27	27
A225W	130	84	82	57	38	37
A225Y	135	103	101	93	69	65

[A] The energy is the $\Delta\Delta G^{fold}$, the change in folding energy upon introduction by the indicated point mutation. The Rosetta 3 and 6 protocols refer to the row 3 and 6 settings of Rosetta_ddg as described in Materials and Methods. None of the three computational protocols allow backbone atoms to move. The color scale acts as a visual guide, with worse effects on stability indicated by a stronger red color.

Table S7 P1' and P2' amino acids in the best and worst substrates docked in omniligase-1. The P1' and P2' amino acids were modeled in DFSKL-P1'-P2'-K molecule that corresponds to the resulting product from P5 to P3'.

**Worst substrates**			**Best substrates**		
P1'	P2'	FRET	P1'	P2'	FRET
Val	Lys	6.07	Ala	Met	61.17
Gln	Asp	5.27	Gly	Met	60.33
Lys	Asp	5.21	Ala	Val	58.82
Tyr	Lys	5.21	Ala	Ile	57.59
Thr	Asp	5.2	Gly	Thr	57.28
Leu	Gly	5.06	Gly	Leu	57.22
His	Ser	4.93	Gly	Ile	56.91
Val	Gly	4.76	Gly	Tyr	56.73
Glu	Asp	4.68	Ser	Leu	56.58
Tyr	Glu	4.48	Ser	Ile	55.33
Val	Asp	3.78	Gly	His	54.02
Lys	Asn	3.42	Asp	Ile	53.61
Val	Glu	3.38	Asp	Leu	53.36

Supplementary Figures

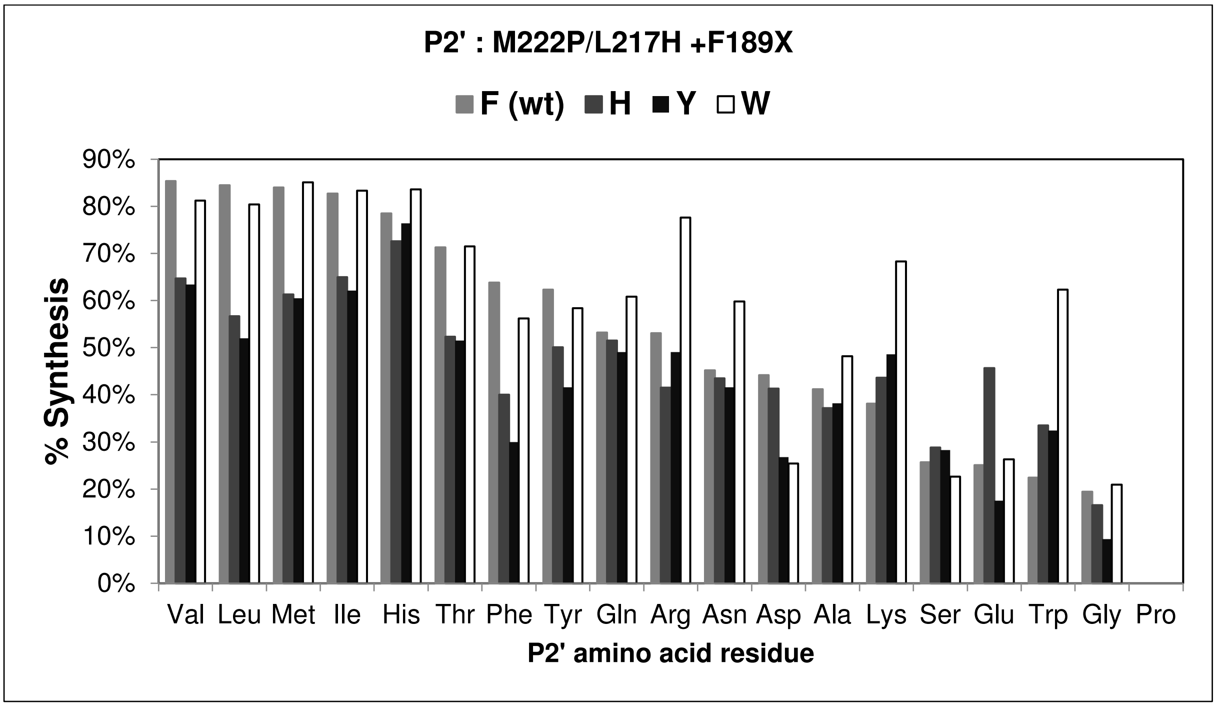

Figure S1. P2’ substrate profile of peptiligase variants Pre-2 and Ptl M222P/L217H/F189W/H/Y. Conversion of Cam-ester starting material was complete in all cases. Formation of synthetic product was determined by LC-MS.

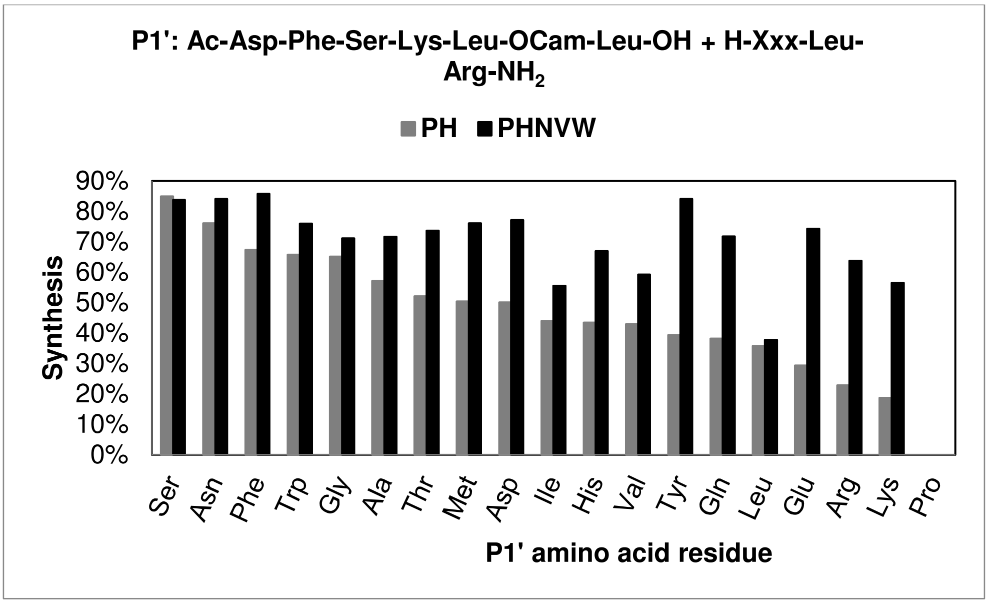

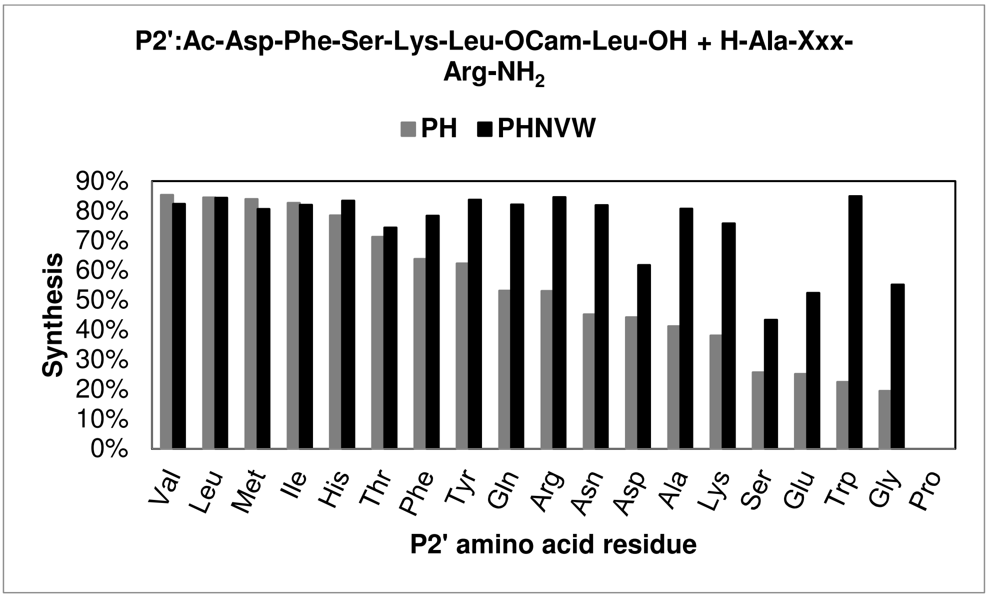

Figure S2. P1′ and P2′ substrate profile of peptiligase variants Ptl M222P/L217H (PH) and omniligase-1 (PHNVW; Ptl M222P/L217H/A225N/I107V/F189W). Conversion of Cam-ester starting material was complete in all cases. Formation of synthetic product was determined by LC-MS.

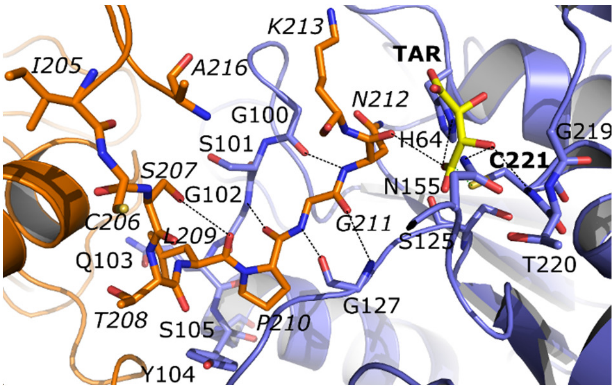

Figure S3. Active site of molecule B of Peptiligase variant Pre-4. The residue names in italics belong to molecule C in orange sticks/cartoon. The other residue names belong to molecule B in slate blue stick/cartoon. The tartrate molecule (TAR) is shown in yellow sticks. The active site Cys221 is shown in bold. Hydrogen bond interactions are shown in black dashed lines.

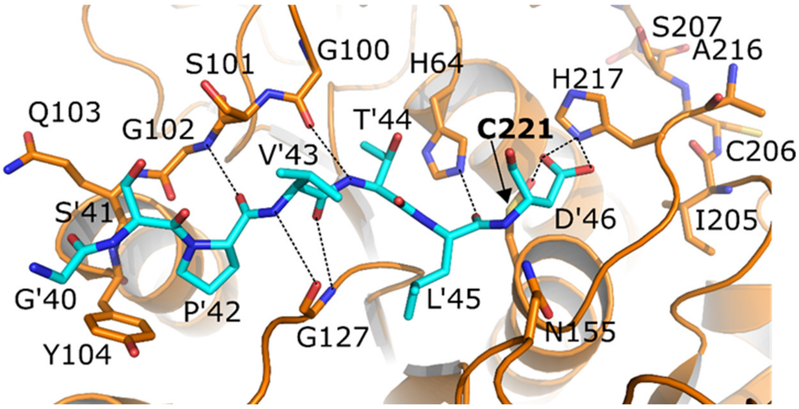

Figure S4. Active site of molecule C of variant Pre-4. The residues belonging to molecule C are shown in orange sticks/cartoon. The residue names labeled with a prime belong to the eglin C fragment (sticks, cyan). Hydrogen bond interactions between the eglin C fragment and the enzyme are shown in black dashed lines.

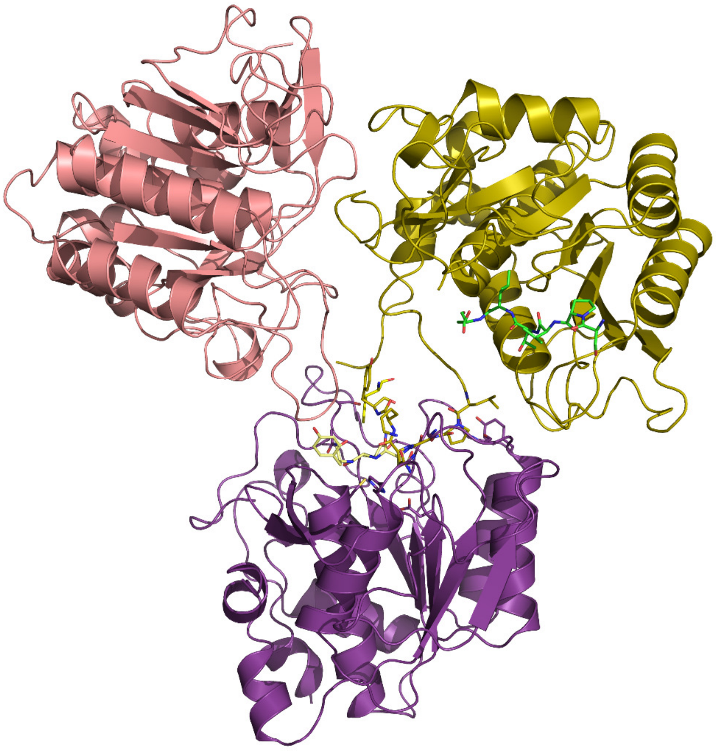

Figure S5. Cartoon representation of variant Pre-5 (pdb code: 7AM7) colored in salmon for molecule A, purple for molecule B and gold for molecule C. The eglin C peptide bound to molecule C is shown in lime green sticks. The loop of molecule C bound in the active site of molecule B is shown in different tints of yellow.

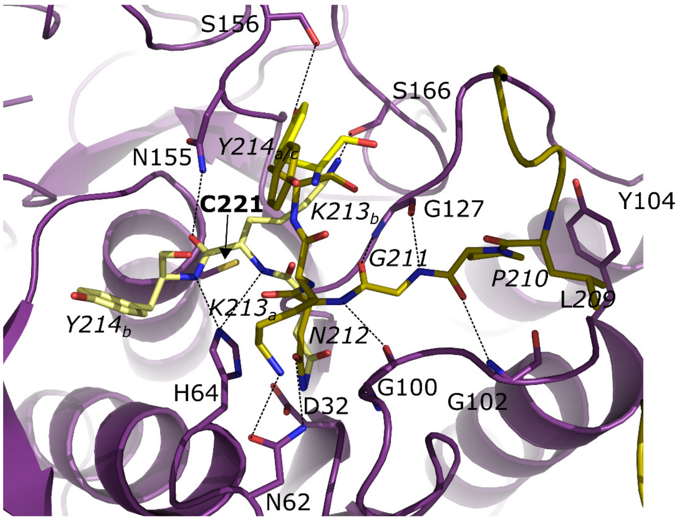

Fig. S6. Active site of peptiligase variant Pre-5 (pdb code: 7AM7) molecule B. The residue names in italics belong to the loop of molecule C in different tints of yellow-gold sticks/cartoon. Different configurations are indicated by subscript letters on the labels and have slightly diverse colors. The other residue names belong to molecule B in slate purple stick/cartoon. Hydrogen bond interactions are shown in black dashed lines.

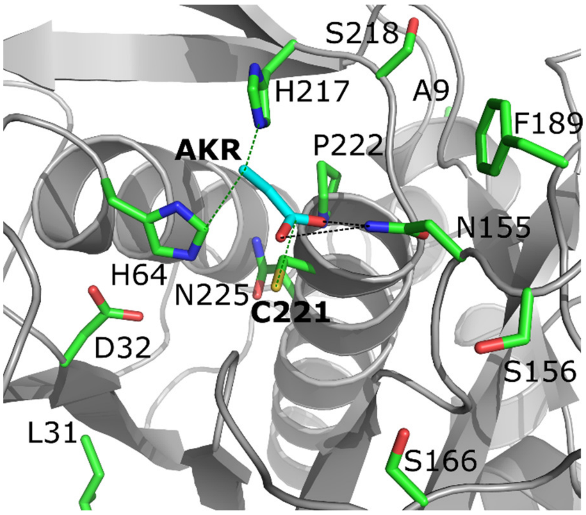

Fig. S7. Active site of Pre-6 variant. The cartoon representation is colored in gray. Mutations from thiol-subtilisin to omniligase are shown in green sticks. In the upper right corner F189, involved in crystal contacts, is shown. The acrylic acid molecule (AKR) is shown in cyan sticks. Hydrogen bonds and other interactions are shown in black and green dashed lines.

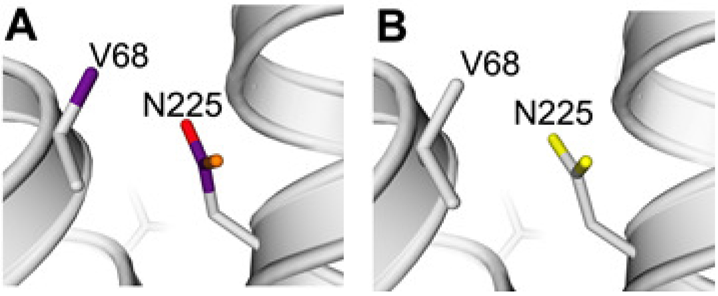

Fig. S8. Strain upon introduction of the A225N substitution is partially released by backbone movements. Panel **A**) N225 modeled within A225 template structure (Pre-2) while fixing the backbone atoms; Panel **B**) idem in an N225 template (Pre-3). The residue labels are at exactly the same horizontal position within the panel to facilitate comparison of the backbone positions. Color code for van der Waals energies: gray, < -5 kJ/mol; orchid, -5 to 0 kJ/mol; yellow, 0 to 2 kJ/mol; orange, 2 to 4 kJ/mol, red > 4 kJ/mol.

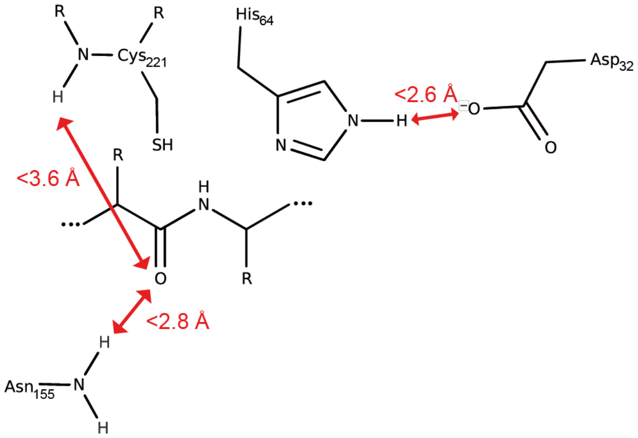

Fig. S9. Geometric criteria applied to select productive binding modes of the peptide.

References

[1] Nuijens, T.; Toplak, A.; van de Meulenreek, M. B. A. C.; Schmidt, M.; Goldbach, M.; Quaedflieg, P. J. L. M. Improved Solid Phase Synthesis of Peptide Carboxyamidomethyl (Cam) Esters for Enzymatic Segment Condensation. Tetrahedron Lett. 2016, 57 (32), 3635–3638. https://doi.org/10.1016/j.tetlet.2016.06.132.

[2] Matthews, B. W. Solvent Content of Protein Crystals. J. Mol. Biol. 1968, 33 (2), 491–497. https://doi.org/10.1016/0022-2836(68)90205-2.

[3] Barlow, K. A.; Ó Conchúir, S.; Thompson, S.; Suresh, P.; Lucas, J. E.; Heinonen, M.; Kortemme, T. Flex DdG: Rosetta Ensemble-Based Estimation of Changes in Protein-Protein Binding Affinity upon Mutation. J. Phys. Chem. B 2018, 122 (21), 5389–5399. https://doi.org/10.1021/acs.jpcb.7b11367.
